# Stearoly-CoA desaturase 1 differentiates early and advanced dengue virus infections and determines virus particle infectivity

**DOI:** 10.1371/journal.ppat.1007261

**Published:** 2018-08-17

**Authors:** Rebekah C. Gullberg, J. Jordan Steel, Venugopal Pujari, Joel Rovnak, Dean C. Crick, Rushika Perera

**Affiliations:** Department of Microbiology, Immunology and Pathology, Colorado State University, Fort Collins, CO, United States of America; The University of Chicago, UNITED STATES

## Abstract

Positive strand RNA viruses, such as dengue virus type 2 (DENV2) expand and structurally alter ER membranes to optimize cellular communication pathways that promote viral replicative needs. These complex rearrangements require significant protein scaffolding as well as changes to the ER chemical composition to support these structures. We have previously shown that the lipid abundance and repertoire of host cells are significantly altered during infection with these viruses. Specifically, enzymes in the lipid biosynthesis pathway such as fatty acid synthase (FAS) are recruited to viral replication sites by interaction with viral proteins and displayed enhanced activities during infection. We have now identified that events downstream of FAS (fatty acid desaturation) are critical for virus replication. In this study we screened enzymes in the unsaturated fatty acid (UFA) biosynthetic pathway and found that the rate-limiting enzyme in monounsaturated fatty acid biosynthesis, stearoyl-CoA desaturase 1 (SCD1), is indispensable for DENV2 replication. The enzymatic activity of SCD1, was required for viral genome replication and particle release, and it was regulated in a time-dependent manner with a stringent requirement early during viral infection. As infection progressed, SCD1 protein expression levels were inversely correlated with the concentration of viral dsRNA in the cell. This modulation of SCD1, coinciding with the stage of viral replication, highlighted its function as a trigger of early infection and an enzyme that controlled alternate lipid requirements during early versus advanced infections. Loss of function of this enzyme disrupted structural alterations of assembled viral particles rendering them non-infectious and immature and defective in viral entry. This study identifies the complex involvement of SCD1 in DENV2 infection and demonstrates that these viruses alter ER lipid composition to increase infectivity of the virus particles.

## Author summary

Dengue viruses are aggressive mosquito-borne viruses causing over 350 million infections annually. There are no antivirals to combat infection and the only vaccine available is suboptimal. Here, we have investigated how these viruses hijack critical metabolic pathways in cells to convert the host environment to support their replicative needs. Specifically, we have identified an enzyme, Stearoyl CoA desaturase (SCD1), that is critical to virus replication and infectious particle formation. SCD1 is responsible for making unsaturated lipids in the cell. When these lipids are incorporated into cellular membranes, they allow these membranes to be more fluid and increase their ability to exchange nutrients between cellular compartments. Inhibition of SCD1 disrupts viral genome replication and blocks structural rearrangements in the virus particles that are required to make them infectious. As a result, SCD1 inhibition causes non-infectious particles to be produced. Additionally, we show that SCD1 enzymatic activity is critical at early stages of virus replication and is shut off during late stages of virus replication.

## Introduction

Phospholipids are critical for membrane structure, function and stability of eukaryotic cells. Specific distributions of lipids within these membranes define their characteristics such as curvature, fluidity, leakiness and the interactions between membranes and membrane-bound protein complexes. A key approach to alter the architecture of a membrane is to incorporate unsaturated fatty acyl chains, to induce curvature and fluidity in a lipid bilayer, altering its functional capacity [1, 2]. Unsaturated fatty acids (UFA) are generated in the cytoplasm and after their initial desaturation they are further elongated, desaturated and shunted towards triglyceride, cholesterol ester or phospholipid synthesis. This initial desaturation event is the rate-limiting step in UFA biosynthesis and is catalyzed at the Δ9 position in the carbon chain by stearoyl CoA desaturase (SCD) [3, 4]. In humans, it has two isoforms: SCD1 is ubiquitously expressed and preferentially converts stearic and palmitic acids into oleic and palmitoleic acids, respectively. SCD5, is restricted to the brain and pancreas [5]. SCD1 is a 40 kD integral membrane protein in the endoplasmic reticulum (ER) and is highly conserved from bacteria to mammals [6]. It regulates the balance between saturated and monounsaturated fatty acids (MUFA) in the cell.

Flaviviruses are obligate intracellular pathogens that hijack lipid metabolic pathways for their energy and substrate requirements. As enveloped viruses, they rely heavily on host phospholipid membranes at every stage of their life cycle and alter the architecture and composition of these membranes to fit their replicative needs [7, 8, 9,10]. Specifically, flaviviruses target membranes of the ER to generate a scaffold for the assembly of viral protein complexes, concentrate substrates required for genome replication, and protect the double-stranded RNA replicative intermediates from detection by the cellular immune response [11]. As a result, the architecture of the ER membrane is altered to form structures known as convoluted membranes (CM), vesicles (Ve) and vesicle packets (Vp) [12, 8, 13]. The CM are considered to be sites for viral protein translation. The Vp/Ve are sites for viral RNA replication [12]. Additionally, these viruses co-opt the ER membrane as a structural component of the virus particle (envelope) and use the ER for virus particle assembly and egress. Embedded in this ER-derived lipid envelope are the viral transmembrane glycoproteins, pre-membrane (prM) and envelope (E). Structural transitions between these proteins are critical for virion maturation and infectivity.

Flaviviral dependence on cellular membranes is reflected in alterations in cellular fatty acid metabolism during infection [14, 15]. This has also been observed for other viruses [16, 17, 18, 19]. The specific physiochemical properties of the required fatty acids and their influence on specific steps of the flaviviral life cycle are not known. In this study, we investigated the importance of fatty acid desaturation on the flavivirus life cycle. We evaluated required enzymes in the UFA pathway using an siRNA library and identified key restrictions that reduced replication of the flavivirus, dengue virus type 2 (DENV2). The enzymatic activity of SCD1 in particular, was required for viral replication and was regulated in a time-dependent manner. SCD1 protein expression levels were inversely correlated with the concentration of viral dsRNA (Replicative Intermediate, RI) in the cell. This modulation of SCD1, coinciding with the stage of viral replication, highlighted its function as an enzyme that controls alternate lipid requirements during early and advanced infections. Loss of function of this enzyme adversely altered the maturation and infectivity of released virions. This study highlights the importance of the UFA biosynthesis pathway in flaviviral genome replication and virion infectivity.

## Results

### Bottlenecks in the UFA biosynthesis pathway control DENV2 replication

**Fig 1:**
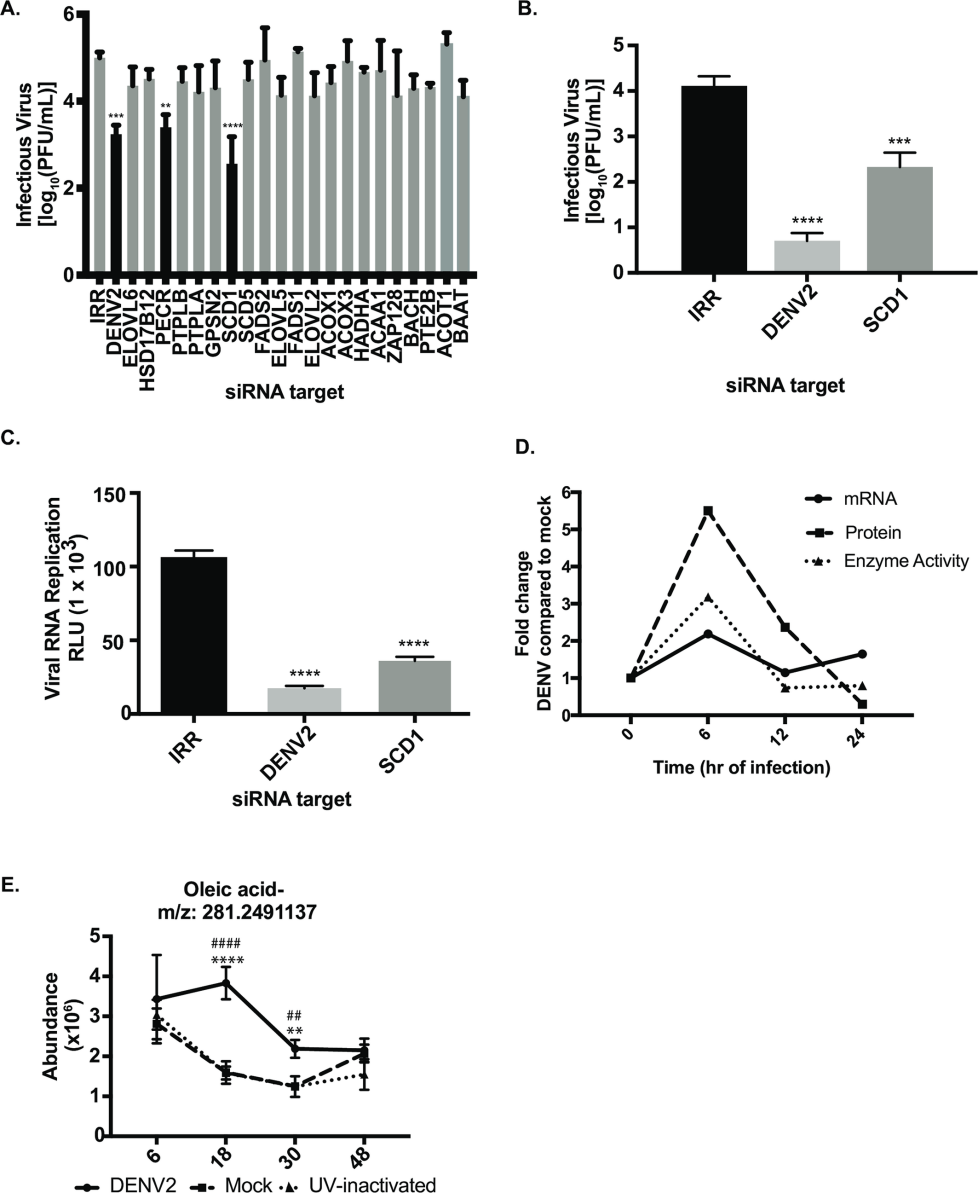
siRNA screen of the UFA biosynthesis pathway indicates that the rate-limiting enzyme is key for DENV2 replication. (A) Huh7 cells were transfected with pools of four siRNAs targeting each gene and infected with DENV2. Infectious virus release was measured by plaque assay. (B) Validation of results using a single siRNA targeting SCD1 as well as indicated controls. (C) siRNAs were electroporated into Huh7 cells along with a luciferase-expressing DENV2 replicon. Viral RNA replication was measured by luciferase expression. (D) Huh7 cells were infected with DENV2 (MOI = 10) and cells were harvested at indicated time points and processed for gene expression, protein levels or enzymatic activity. Fold change in SCD1 mRNA in virus-infected cells was compared to mock-infected cells. qRT-PCR results are normalized to GAPDH. The same cells were flash frozen to preserve active enzymes. Cytoplasmic extracts were prepared and run on a western blot and probed for SCD1. Protein levels were normalized to actin. For enzyme activity measurements, TLC was carried out to measure conversion of ^14^C-labeled Stearoyl-CoA to ^14^C-labeled-oleic acid. The quantification of these 3 assays (mRNA, protein and SCD1 activity) is shown here. Results from a single representative experiment are presented. Three independent biological replicates showed similar temporal trends. (E) Huh7 cells were infected with DENV2, a UV-inactivated DENV2 or mock-infected. Five biological replicates were included at each time point. Metabolites were extracted and untargeted LC-MS was performed to measure the abundance of oleic acid at each time point. SCD1: stearoyl CoA desaturase, IRR: irrelevant siRNA (without a biological target for the siRNA sequence), DENV2: siRNA against dengue virus, serotype 2 genome. (A-C: one-way ANOVA with multiple comparisons tests: ** = p<0.05, *** = p<0.001, **** = p<0.0005, For E: DENV2 vs. mock: ** = p<0.001, **** = p<0.0001 and DENV2 vs UVI: ## = p<0.001, #### = p<0.0001).

We hypothesized that enzymes in the UFA biosynthesis pathway are important for the DENV2 lifecycle and interrogated this pathway with siRNAs (S1 Table) to determine effects of their transient knock-down on viral replication (Fig 1 and S1 Fig). An irrelevant siRNA (IRR) controlled for off-target effects of siRNA treatment, while an siRNA targeting the DENV2 genome was a positive control for reduction in viral replication. We identified two enzymes in this pathway that represent host-viral interaction points, SCD1 and peroxisomal trans-2-enoyl-coA reductase (PECR) (Fig 1A). These observations were made in two human cell lines (Huh7 and A549), yielding similar effects on viral replication (S1A and S1B Fig). Cytotoxic effects of siRNA treatment were not significant (S1C–S1E Fig).

We found that knockdown of SCD1 expression significantly reduced DENV2 replication in all cell lines and conditions tested when compared to an irrelevant siRNA (Fig 1A, B and 1C and S1A and S1B Fig). Since the initial screen was carried out with a pool of four siRNAs against SCD1, we also validated the results with a single siRNA against SCD1 and showed a similar reduction in virion release (Fig 1B). This siRNA was further tested against a luciferase expressing DENV2 replicon [14], and we found that viral RNA replication was significantly reduced (Fig 1C). Therefore, the effect of SCD1 knockdown on the release of infectious virus is at least partly mediated at the RNA replication step. We confirmed that siRNA treatments were effective at reducing SCD1 mRNA and protein levels using qRT-PCR and western blot analyses. The SCD1 mRNA expression was reduced by 90% and protein levels were below the level of detection (S1F and S1G Fig). These data suggest that UFA synthesis is critical for DENV2 replication.

### SCD1 gene expression and activity are elevated early post-DENV2 infection

Based on the above observations, we hypothesized that DENV2 requires a stable or increased level of the SCD1 enzyme and its products to regulate the cellular lipid repertoire for its replicative advantage, and that the activity of this enzyme may be controlled by viral infection. Since SCD1 expression is regulated at the transcriptional level [20], we first examined SCD1 mRNA levels in infected cells and found them to be increased at early time-points post infection compared to mock-infected cells (Fig 1D). To ensure that the protein is translated and active during viral replication, we quantitatively examined the enzymatic activity of SCD1 in DENV2-infected cells over time. Consistent with the mRNA expression profile at the early time points, we found that cells infected with DENV2 had an initial increase in SCD1 activity, as measured by the conversion of radiolabeled stearic acid to oleic acid, as early as 6hr post infection (Fig 1D). This coincides with early replication and translation of the viral genome. Also, consistent with changes in its mRNA profile, later during infection we found a decrease in SCD1 expression and activity. SCD1 is the only enzyme that can produce oleic acid. Our observations suggest that DENV2 infection specifically up-regulates SCD1 activity early in infection to expand the pool of MUFAs available for its replicative needs. Since mRNA, protein and enzymatic activity of SCD1 measured above collectively contribute to producing oleic acid, we measured oleic acid using high-resolution, liquid chromatography-mass spectrometry (LC-MS). Specifically, we observed that pools of oleic acid were increased in Huh7 cells infected with DENV2 compared to two controls; UV-inactivated DENV2 exposed cells and mock-infected cells (Fig 1E). The UV-inactivated DENV2 is capable of binding to and entering cells, but cannot replicate [15]. These data indicate that actively replicating virus is required to activate SCD1.

### SCD1 is found within early centers of viral replication

**Fig 2:**
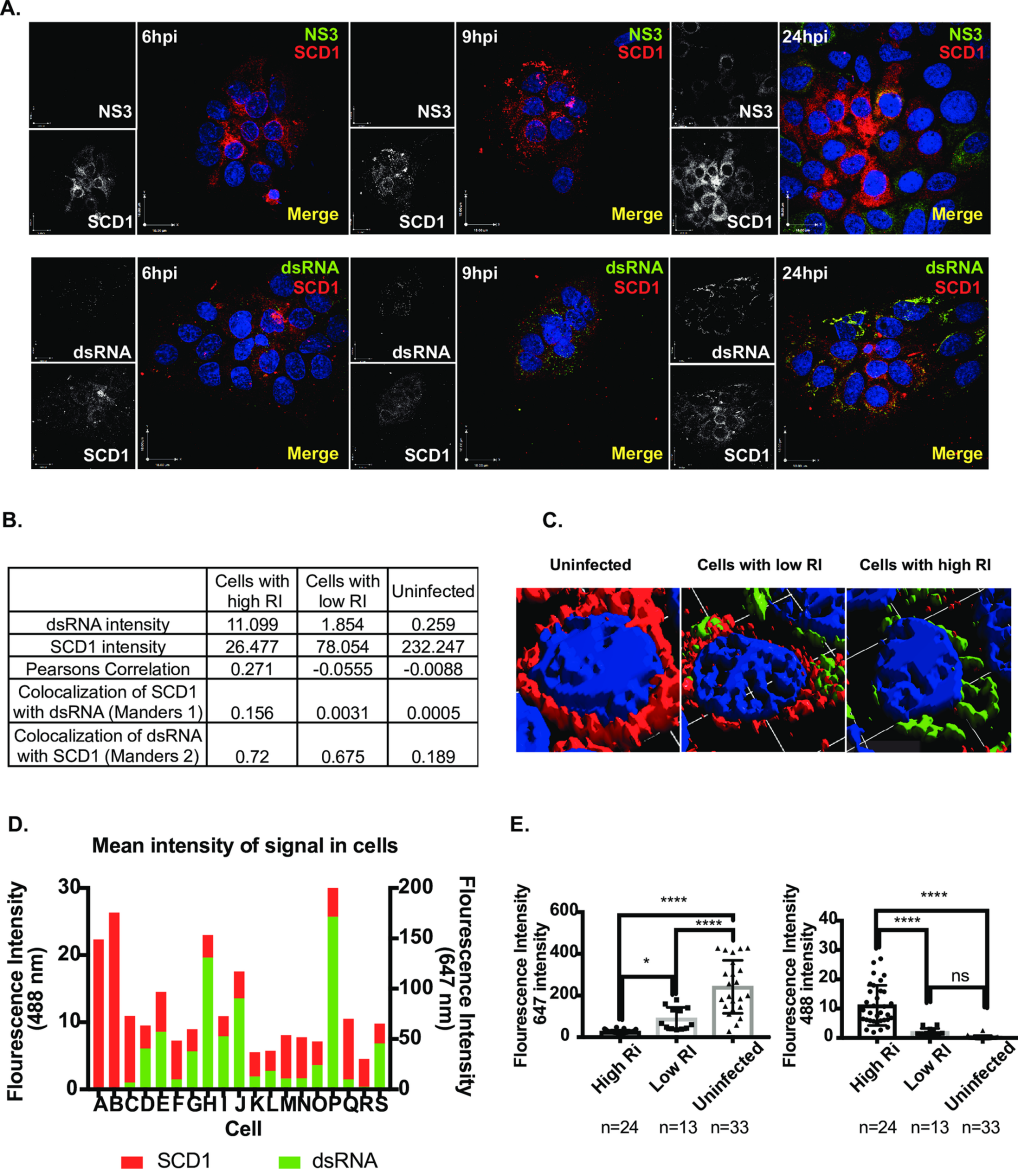
SCD1 protein expression is modulated with level of viral replication. (A) Immunofluorescence analysis of Huh7 cells uninfected or infected with DENV2 at three time points. Viral protein NS3 or dsRNA (488nm, green), SCD1 (647nm, red). (B) Cells were classified as uninfected, low or high viral RI, based on their dsRNA (488 nm, green) signal. A summary of the mean intensities of dsRNA and SCD1 in the 3 cell populations with co-localization coefficients: Pearsons global correlation and Manders correlation M1 and M2. (C) 3-D reconstructions of 3 representative cells at 36hr showing dsRNA and SCD1. (D) Mean fluorescent intensity of dsRNA and SCD1 signals was measured in each cell of a representative image frame at 36hr. (E) Mean fluorescent intensity of each cell in multiple images is shown as a dot plot. The average 488 nm (dsRNA, green) signal and 647 nm (SCD1, red) signal for each group of cells is plotted as a bar graph. (ns = not significant, ** = p<0.005, *** = p<0.001, **** = p<0.0001, from a one-way ANOVA with a multiple comparisons tests), hpi: hours post-infection, RI: Replicative Intermediate.

Previous studies with DENV2 have shown that lipid biosynthetic enzymes (such as fatty acid synthase, FAS) are recruited to viral replication complexes, to increase local synthesis of lipids at sites of viral RNA replication and virus assembly [14]. Since SCD1 is immediately downstream of FAS in the biosynthetic pathway, we investigated whether SCD1 was also re-localized to viral replication complexes during DENV2 infection. We processed mock-infected or DENV2-infected cells at 6, 9 and 24hr for immunofluorescence studies, using antibodies against DENV2 NS3, dsRNA (RI) and human SCD1 (Fig 2). Interestingly, unlike what we previously observed for FAS in Huh7 cells [14], there is a temporal progression in marker distribution (best observed in Fig 2A, 24hr dsRNA and SCD1 panel). Uninfected cells show normal SCD1 signal (S2A Fig and Fig 2C), while cells with a low level (early) infection show that SCD1 can be found within viral replication complexes. Using Manders co-localization values [21, 22], we can see a correlation between RI and SCD1 in all infected cells (Fig 2B). However, cells with high levels of viral RI (late infection) show very low levels of SCD1 (Fig 2C). Quantification of the mean fluorescent intensity in a representative image of these cells demonstrated an inverse correlation between levels of SCD1 and viral markers (Fig 2D and 2E). These data correspond with our findings of SCD1 expression and enzymatic activity, where Huh7 cells with high levels of RI at late time points of infection have reduced levels of SCD1. The initial spike in SCD1 activity from SCD1 localized around viral replication and assembly sites early during infection likely generates concentrations of oleic acid sufficient for the metabolic needs throughout replication. The specificity of the SCD1 antibody was tested in SCD1 siRNA-treated cells (S2B and S2C Fig). We did not see this distribution of SCD1 in DENV-infected A549 or human embryonic lung (HEL) cells. Rather we see a uniform co-localization of SCD1 and NS3 (S2D and S2E Fig). This suggests that changes in SCD1 expression levels are cell-type specific.

### Inhibition of SCD1 disrupts replication of all DENV serotypes and other enveloped viruses

**Fig 3:**
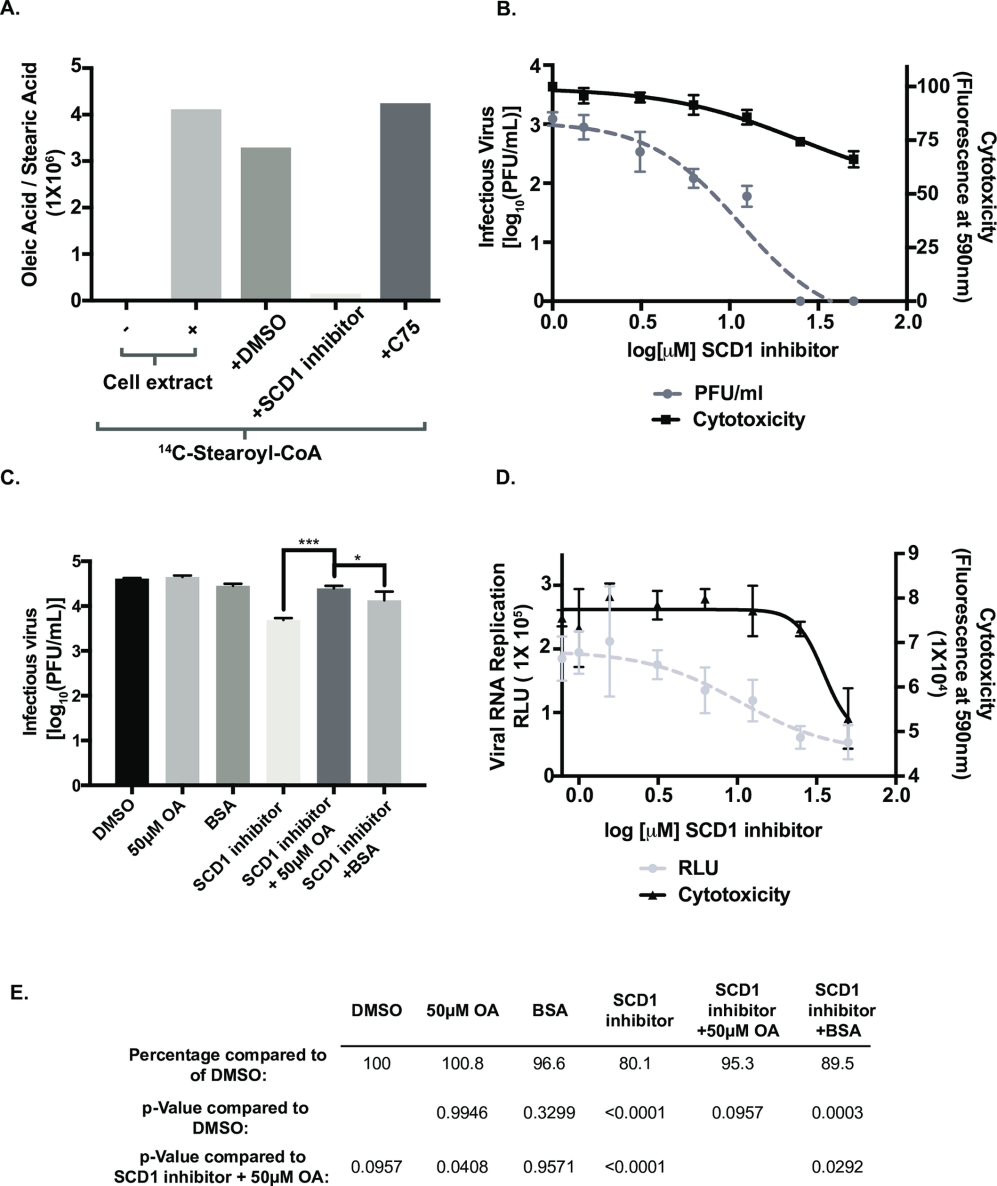
Oleic acid supplementation following inhibition of SCD1 rescues viral replication. (A) The production of ^14^C-labeled oleic acid and stearic acid in uninfected cell extracts were quantified and compared across indicated conditions. (B) Infectious virus release from Huh7 cells infected with DENV2 (MOI = 0.5) and treated with the indicated concentrations of the SCD1 inhibitor for 24hr. Cytotoxicity was also measured. (C) Huh7 cells were infected with DENV and treated with 10 μM SCD1 inhibitor and 50 μM oleic acid conjugated to BSA or indicated controls. At 24hr post infection virus was collected and titrated by plaque assay. (D) Huh7 cells were electroporated with RNA from a DENV2 luciferase-expressing replicon and treated with the indicated concentrations of the SCD1 inhibitor. RLU and cytotoxicity was measured at 24hr. (E) The virus titers in part C were quantified and their percent compared to DMSO to better represent the levels of inhibition and rescue. (* = p = 0.05, *** = p<0.001, from a one-way ANOVA with a multiple comparisons test).

We used a pharmacological inhibitor to characterize the enzymatic requirement for SCD1 during DENV2 replication. The piperidine-aryl urea-based inhibitor, A939572, which we will refer to as the SCD1 inhibitor has been shown to be effective [23]. We tested the SCD1 inhibitor in our activity assay and found that it abolished the formation of oleic acid (Fig 3A). In DENV-infected cells, SCD1 inhibition resulted in a dose-dependent reduction in viral titers (up to 2 logs) without significant toxicity (Fig 3B). Analysis of the effectiveness of the SCD1 inhibitor as an antiviral compound gave a therapeutic index of 2.1. The SCD1 inhibitor was also effective against DENV2 replication in A549 cells but it had no effect on mosquito cells (S3A and S3B Fig), suggesting that the Δ9 desaturase in arthropods may differ from the mammalian version.

Inhibition of SCD1 halts the desaturation of stearic acid (C18:0), acid leading to a decrease in cellular concentrations of oleic acid (C18:1). Oleic acid is a key building block for more complex phospholipids, cholesterol esters and triglycerides that function as constituents of cellular and virus-induced membranes. We hypothesized that addition of exogenous oleic acid would rescue the effect of SCD1 inhibition on virus replication. To accomplish this we added oleic acid conjugated to BSA in serum free medium combined with the SCD1 inhibitor and measured viral replication. We found that addition of oleic acid restored viral replication, implying that the product of SCD1 enzymatic activity is critical for DENV2 replication (Fig 3C). The rescue was not complete (95.3%, compared to the rescue with DMSO), likely because exogenous fatty acids have many destinations in the cell and are often shunted to β-oxidation (Fig 3E) [24]. Therefore, they may be minimally incorporated into the ER where they could be used for virus replication.

**Fig 4:**
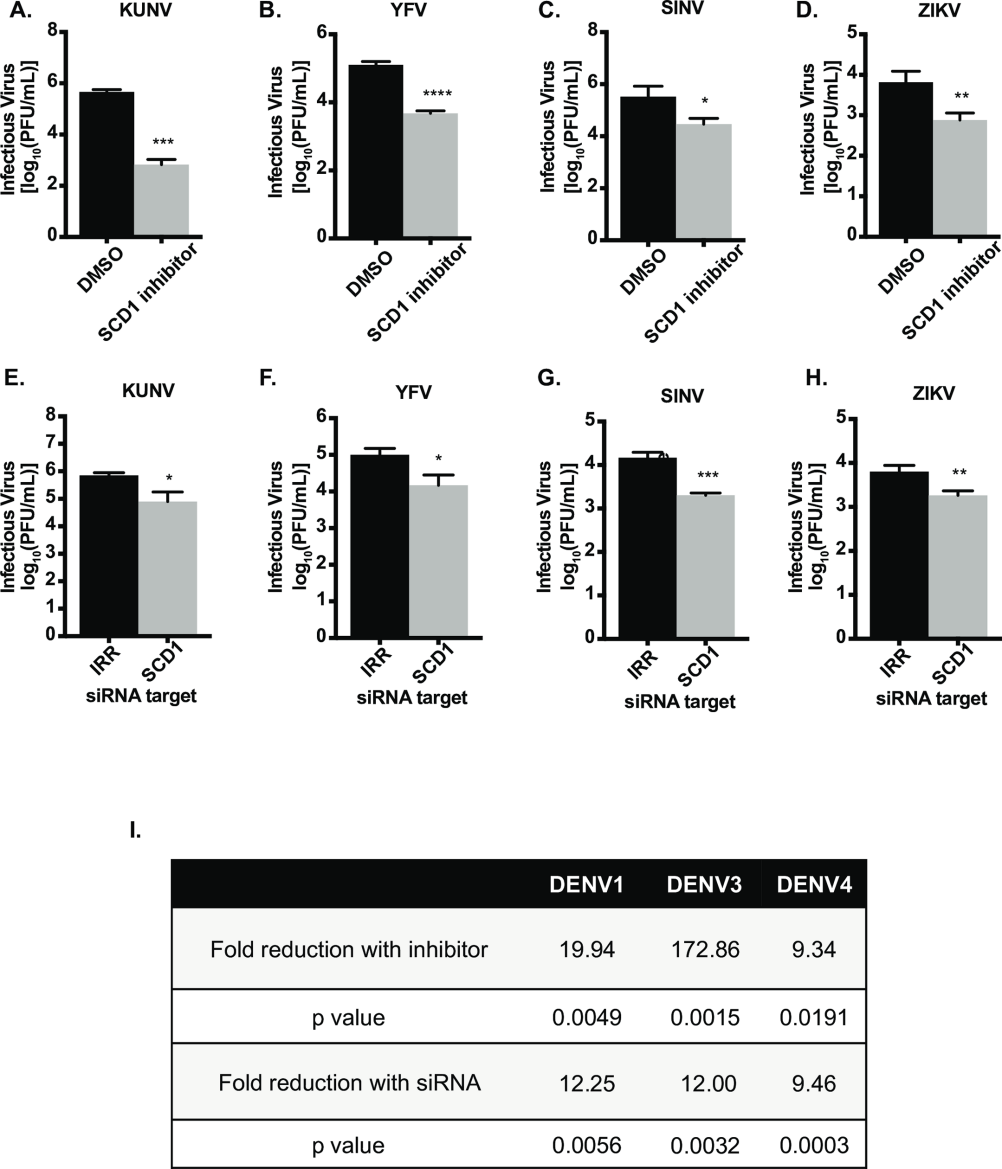
Inhibition and knockdown of SCD1 reduces replication of multiple enveloped viruses. Huh7 cells were infected with (A) KUNV (MOI = 0.1), (B) YFV (MOI = 0.1), (C) SINV (MOI = 0.01) or (D) ZIKV (MOI = 0.5) and treated with 10μM of the SCD1 inhibitor or DMSO. (E-H) Huh7 cells were transfected with IRR or SCD1 specific siRNA and infected after 48hr of knockdown with (E) KUNV (MOI = 0.1), (F) YFV (MOI = 0.1), (G) SINV (MOI = 0.01) or (H) ZIKV (MOI = 0.5). Supernatants were collected at 24hr post infection for KUNV, YFV and ZIKV, and at 8hr post infection for SINV according to the time point for maximum viral replication. Virus release was quantified by plaque assay on BHK cells. (I) Summary of results for DENV serotypes 1, 3 and 4. Inhibitor and siRNA analyses were done similar to the previous experiments. Unpaired t-tests were performed for all experiments indicating significant reduction in viral replication with inhibition or knockdown of SCD1. (* = p<0.05, ** = p<0.01, *** = p<0.0005, **** = p<0.0001) compared to control).

Next we tested the SCD1 inhibitor for its effects on the replication of other enveloped, mosquito-borne viruses. We used a non-cytotoxic concentration that was effective against DENV2 and found that the replication of all other DENV serotypes and Kunjin virus (KUNV), yellow fever virus (YFV), Zika virus (ZIKV) and Sindbis virus (SINV) was significantly reduced (Fig 4A–4D and 4I). To confirm that this was not due to off-target effects of the inhibitor, we knocked down SCD1 with siRNA and found similar effects on viral replication (Fig 4E–H and 4I). These data indicate a common need for SCD1 enzymatic activity and incorporation of MUFAs into complex lipid species to aid in the replication of enveloped viruses.

To further characterize the impact of SCD1 inhibition on the DENV2 life cycle, we carried out a time of addition experiment. We found that addition of the inhibitor prior to infection or during attachment followed by removal of the inhibitor had no impact on viral replication (S4A and S4B Fig). However, addition of the inhibitor at any time point after infection resulted in a decrease in viral replication compared to the vehicle control (S4C Fig). Addition of the siRNA after 24 hr of viral replication, however had no impact on viral replication (S4D and S4E Fig). Taken together SCD1 activity is important for viral replication at multiple stages of the virus life cycle, but may be more important at the early time points and less critical at later time points.

### SCD1 activity is critical for infectious virus release

**Fig 5:**
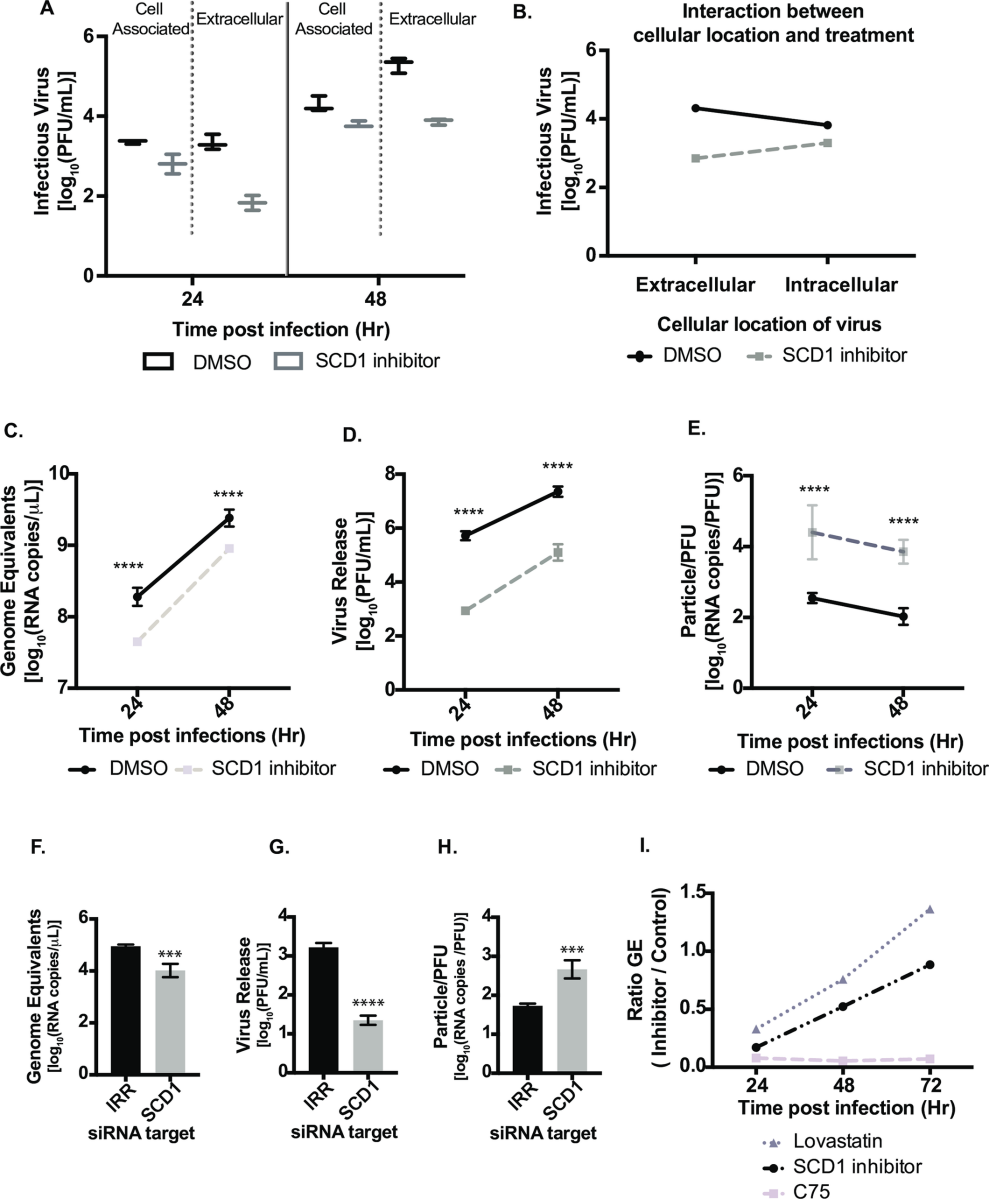
Inhibition of SCD1 impacts viral particle infectivity. Huh7 cells were infected with DENV2, MOI = 1 and treated with SCD1 inhibitor or DMSO. (A) Virus in supernatants (extracellular) and cell-associated virus were collected at 24 and 48hr and quantified by plaque assay. (B) Statistical interaction plot of data from A. The non-parallel lines indicate interaction between the cellular location of the virus and the treatment. A three-way ANOVA confirmed this interaction with a p = 1.600e-06. (C-E) Huh7 cells were infected with DENV2 and treated with the SCD1 inhibitor or DMSO. Supernatants were collected at 24 and 48hr. Virus was quantified by plaque assay. Virus RNA was extracted from the same samples for qRT-PCR analysis. (C) GE were determined by qRT-PCR using a standard curve of viral RNA copies. (D) The titer of the viruses at each time point as determined by plaque assay. (E) The particle:pfu ratio was calculated by dividing the RNA copies/mL by the PFU/mL from C and D. (F-H) Huh7 cells were transfected with siRNAs for SCD1 or an irrelevant target (IRR) and incubated for 48hr. They were then infected with DENV2 (MOI = 0.1) and supernatant was collected after 24hr. Virus was quantified by plaque assay. Viral RNA was extracted from the same samples for qRT-PCR analysis. (F) GE were determined by qRT-PCR using a standard curve of viral RNA copies. (G) The titer of the viruses was determined at each time point by plaque assay. (H) The particle:pfu ratio was calculated by dividing the RNA copies/mL by the PFU/mL from F and G. (I) Comparison of GE in cells treated with the SCD1 inhibitor to those treated with lipid synthesis inhibitors, C75 and Lovastatin. [Inhibitor: (Genome equivalents/mL) / DMSO: (Genome equivalents/mL)]. (*** = p<0.005, **** = p<0.0001). GE: Genome equivalents.

Having determined that SCD1 is important for the DENV2 life cycle, we investigated its requirement at specific stages of viral replication. Using a viral replicon, we found that inhibition of SCD1 reduced DENV2 RNA replication (Fig 3D). Viral RNA replication and assembly are tightly coordinated [25]. We examined the release of infectious virus particles by quantifying the intra- and extracellular virus at 24 and 48hr post infection and found that intra- and extracellular virus from untreated cells had equivalent titers at 24hr with an increase in extracellular virus titer at 48hr (Fig 5A). However, virus grown in the presence of SCD1 inhibitor lagged in release of extracellular infectious virus compared to intracellular virus at 24hr. Titers reached equivalence at 48hr but were below those of untreated controls (Fig 5A). Three-way ANOVA confirmed a significant interaction between inhibitor treatment and intracellular vs. extracellular virus location (Fig 5B; p = 1.600e-06 for the interaction term). These analyses indicate that inhibition of SCD1 affects the release of infectious virus.

We next investigated the effect of SCD1 inhibition on the ratio of infectious and non-infectious virus particles released. We measured the ratio of total particles released to infectious-particles released (the specific infectivity) for virus grown in cells exposed to the SCD1 inhibitor compared to untreated cells. We found a small but significant reduction in total particles released from SCD1-inhibited cells as measured by genome equivalents (GE) (Fig 5C). However, the titer of infectious particles (as measured by plaque assay) was reduced at both time points by almost 100-fold, similar to our previous results (Fig 5D), and indicated a reduction in the specific infectivity of virus released from inhibitor-treated cells (Fig 5E). Hence, when cells are treated with the SCD1 inhibitor, the total numbers of viral particles released was only slightly lowered compared to control cells, but fewer of these viral particles were infectious (Fig 5C–5E). Similar results were observed when loss of function studies were carried out using an siRNA against SCD1 (Fig 5F–5H). This observation was also confirmed with ZIKV, another flavivirus that also showed reduced particle release from Huh7 cells treated with the SCD1 inhibitor (S5A Fig). This was not observed in C6/36 mosquito cells treated with SCD1 inhibitor (S5B Fig). For DENV2, GE in virus particles released from SCD1 inhibitor-treated Huh7 cells were compared to GE from cells treated with two other lipid synthesis inhibitors, C75 (inhibits FAS) and Lovastatin (inhibits cholesterol synthesis) (Fig 5I), that had previously been shown to be effective against DENV2 [14, 15, 26]. We observed a defect in the release of infectious particles with all treatments (S5C and S5D Fig), however, C75 treatment resulted in a larger decrease in the GE ratio compared to the other treatments (Figs 5F and compare S5E to S5F). FAS activity is critical for DENV2 genome replication [14], while cholesterol (altered by Lovastatin) is critical for maturation of DENV2 and generation of infectious particles [27]. Inhibition of SCD1 was similar to inhibition of cholesterol biosynthesis (Figs 5 and S5), further demonstrating its significance in the virus life cycle.

### Virion infectivity is altered following SCD1 inhibition

**Fig 6:**
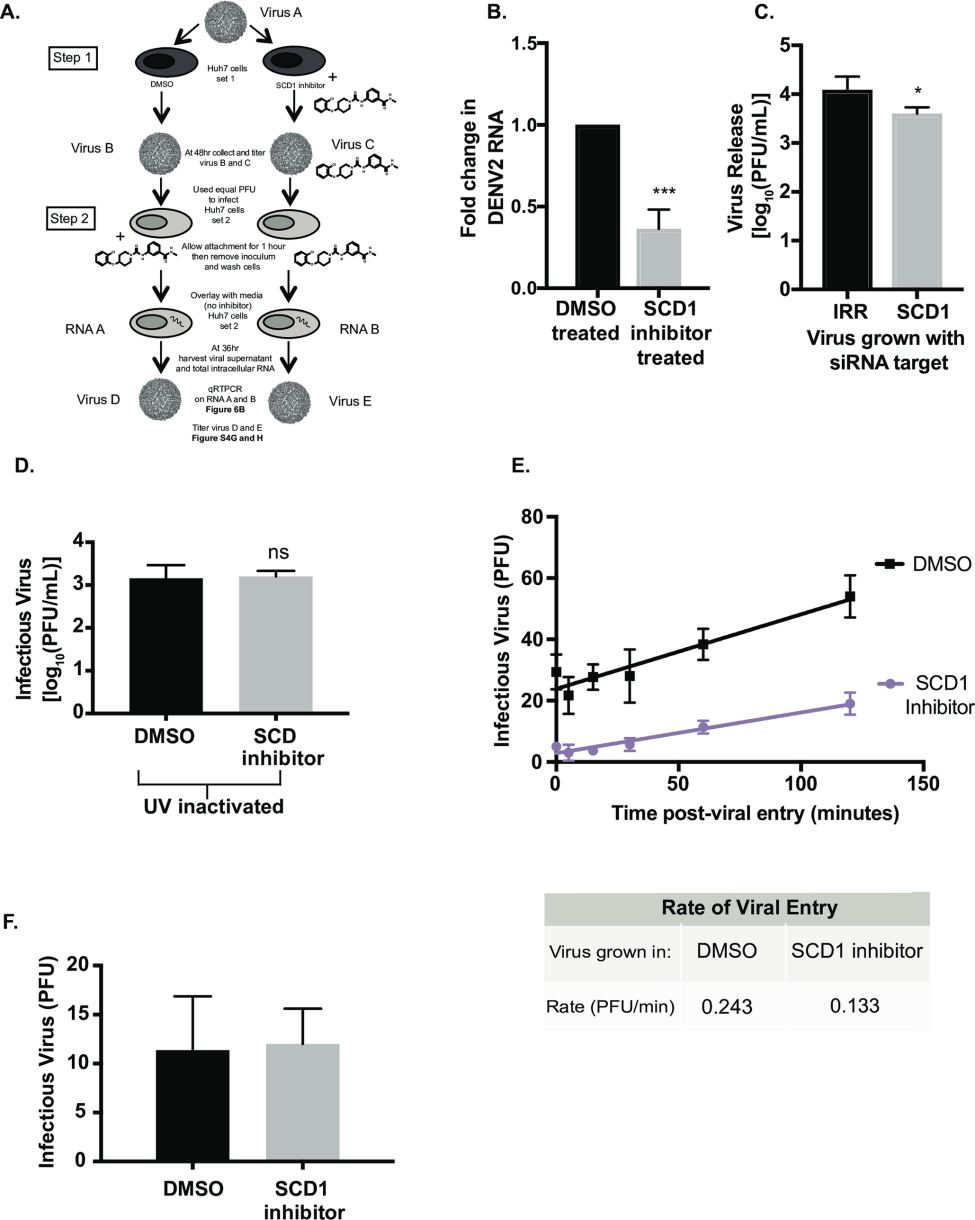
Infectious particles grown in the presence of the SCD1 inhibitor are slower to infect new cells. **(**A) Schematic of the experimental design: Step 1: Huh7 cells (set 1) were infected with DENV2, MOI = 3 (Virus A) and treated with DMSO or the SCD1 inhibitor. At 48hr the virus was titrated (Virus B and C). Step 2: This virus was used to infect naïve Huh7 cells (set 2) at a MOI of 0.1. The concentration of inhibitor remaining in viral supernatant C was mimicked by adding inhibitor to viral supernatant B during attachment. Cells were washed and overlaid with media without inhibitor and incubated for 36hr. Total RNA (RNA A and B) and virus supernatant (Virus D and E) were collected. (B) Viral RNA copies (RNA A and B) from Huh7 cells (set 2) were measured by qRT-PCR. The fold change of viral RNA copies in RNA B compared to RNA A is shown. (C) Huh7 cells with siRNA knockdown of SCD1 or an irrelevant control were infected with DENV2 (MOI = 0.1) for 48hr. Virus supernatant was collected and titrated. This virus was used to infect new Huh7 cells with an equal MOI (0.3). Virus was grown for 48hr and the supernatant was titrated. (D) Supernatants were collected from DENV infected cells with or without the inhibitor and UV-inactivated. WT DENV2 was then diluted in these UV-treated supernatants and used to infect new cells with an equal MOI. (E) Virus grown with the SCD1 inhibitor was again titrated and used to infect BHK cells with 100pfu/well. Attachment was allowed to occur at 4°C for 2hr, the temperature was shifted to 37°C and the cells were treated with acid glycine at the indicated time points after infection to inactivate un-internalized virus. Cells were overlaid with agarose and plaques were counted at 6 days. A linear regression was performed. The slope of the entry of the virus grown in the presence of the SCD1 inhibitor was 0.14PFU/min and DMSO was 0.27 PFU/min. (F) Huh7 cells were infected with DENV2 (MOI = 3) and treated with SCD1 inhibitor or DMSO. Supernatants were collected at 48hr, RNA was extracted, viral RNA copies were measured by qRT-PCR. Equal RNA copies were transfected into BHK cells to allow plaques to form. (ns = not significant, *** = p<0.001, from a two-tailed t-test).

We evaluated the virions released from inhibitor-treated cells for defects in infectivity. DENV2 was passaged in Huh7 cells in the presence of the SCD1 inhibitor or DMSO, and released virus particles were collected, titrated, and used to infect new Huh7 cells in the absence of inhibitor (Fig 6A). During adsorption, the same concentration of inhibitor (10μM) was added to control supernatants to mimic remaining, un-metabolized inhibitor in the treated-cell supernatant. Virus isolated from inhibitor-treated cells had reduced infectivity compared to virus from control cells as determined by intracellular viral RNA (Fig 6B) and a reduction in released infectious virus (S5G Fig). This was confirmed in A549 cells (S5H Fig). We confirmed this result with loss of function studies using an siRNA specific to SCD1 (Fig 6C). To control for possible interference by defective particles in drug-treated cell supernatants, we UV-inactivated virus in supernatants from drug-treated and control cells and added it to equal titers of untreated virus stock to observe possible inhibition of infection. We found no difference in the resulting production of infectious virus, indicating that defective particles from cells treated with the SCD1 inhibitor did not interfere with subsequent virus infection (Fig 6D). Therefore, the defect in initiating a second round of infection is unique to virus particles released from cells lacking SCD1 activity.

We examined the early kinetics of viral infection with the virus from inhibitor-treated cells versus virus from control cells. We used a viral entry assay to determine the amount of virus internalized or endocytosed at given time points after attachment. Virus still external to the cell was inactivated at the indicated time point. The virus from cells treated with SCD1 inhibitor was slower to enter new cells (0.133 PFU/min versus 0.243 PFU/min for the control virus; Fig 6E). This defect was found both in the rate at which virus entered cells as well as the number of virus particles that entered the cells at equal titer of infection. Inhibition of SCD1 lowers the quantity of and changes the characteristics of infectious particles. These data indicate that there is an attachment or fusion defect that is generated in cells with decreased SCD1 enzymatic activity.

To determine if the defect was in the physical structure of the virus particle (either the prM/E glycoprotein shell or virion lipid envelope) or in the genome encapsidated within virus particles released from SCD-inhibited cells, we isolated and transfected the RNA from these virus particles into BHK cells and measured the ability of the RNA to initiate infection. We found that both viral RNA populations were able to initiate infections with the same efficiency (Fig 6F).

### Inhibition of SCD1 results in release of immature virions

These data suggest that there is a change to the physical structure of the virion when it is released from cells lacking SCD1 activity. We initially tested if the virions were less thermally stable when grown in the presence of the SCD1 inhibitor compared to controls. The virus from SCD1-inhibitor treated and control-infected cells were diluted to the same infectious virus concentration, heated to the indicated temperatures (S6A Fig), and titrated. Nonlinear regression demonstrated that the control virus lost infectivity at 44.03°C and the SCD1 inhibitor-treated virus lost infectivity at 43°C. An F-test to determine the difference between the two models indicated no significant difference (S6A Fig). We also carried out freeze/thaw cycles on virus samples and measured infectivity and found that control virus maintained its infectivity for 6 or more freeze/thaw cycles, but virus grown in the presence of the SCD1 inhibitor lost its infectivity after 3 freeze/thaw cycles (S6B Fig). This effect was also observed with Zika virus grown in the presence of the SCD1 inhibitor (S6C Fig). We limited the defect in SCD1 inhibitor-treated virus infectivity to the structure of the virion envelope, which is more susceptible to freeze-thaw transitions than the control.

**Fig 7:**
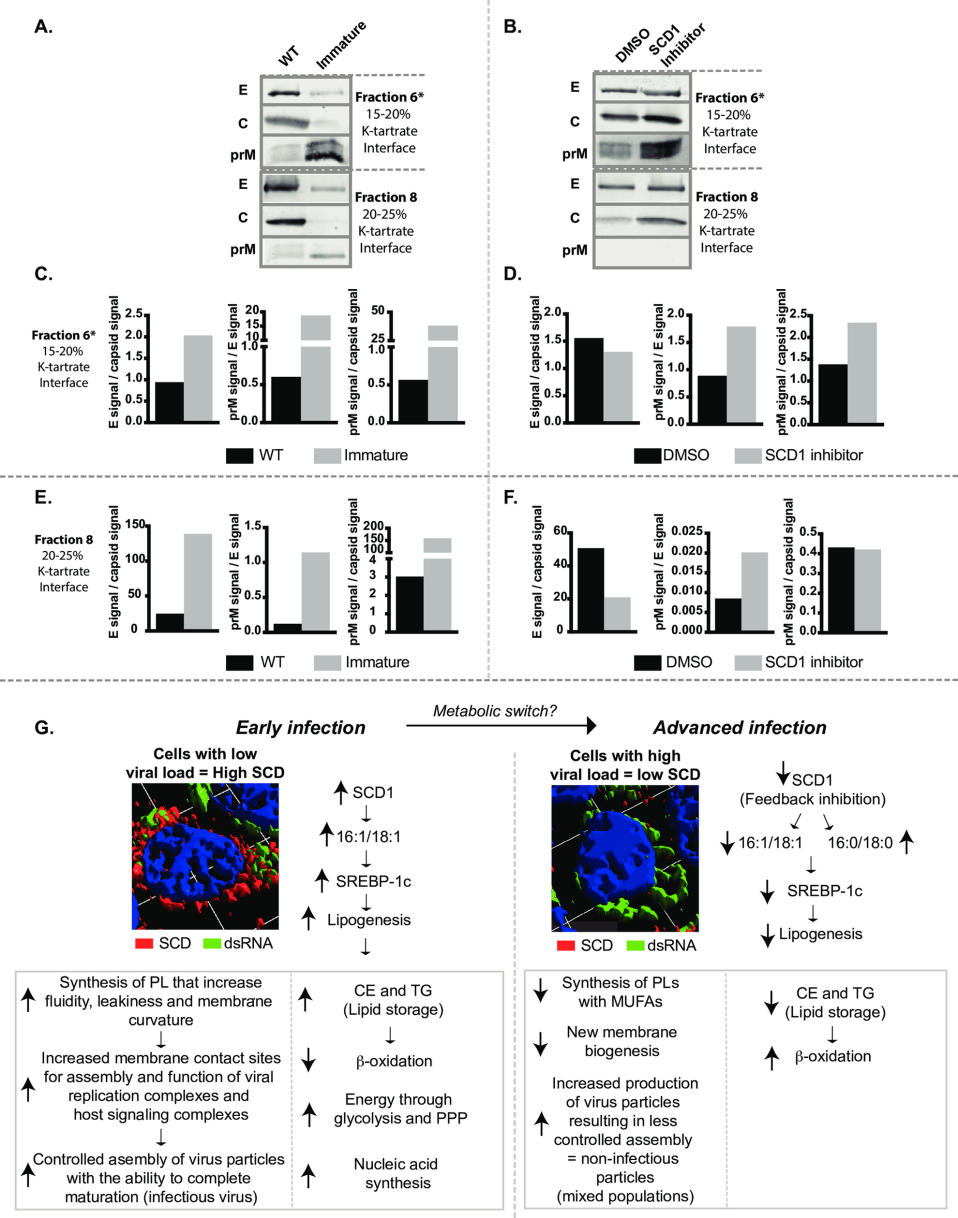
Inhibition of SCD1 Impairs viral maturation. Huh7 cells were infected with DENV2 and left untreated (WT), treated with 20 mM NH_4_Cl (immature), 10 μM SCD1 inhibitor, or vehicle (DMSO). (A) and (B) At 72 hr post-infection virus purified by density gradient sedimentation. Bands (fractions 6 and 8) where the highest concentration of genomes were observed (and previously known to have virus particles) were buffer-exchanged, concentrated and processed by western blot with antibodies for envelope, capsid and prM proteins. (C-F) The relative quantification of the viral glycoproteins prM and E in fractons 6 and 8. (G) A proposed model for metabolic changes involving SCD1 that differentiates the metabolic state in early versus advanced DENV2 infections. E: envelope protein, c: capsid protein, prM: pre-membrane protein, CE: cholesterol Esters, TG: triglycerides.

During infection, cells produce a range of structurally diverse particles with varying levels of infectivity. The main structural classes are immature, partially mature or fully mature and they are defined by the amount of uncleaved prM protein retained on the virion [28, 29]. Although, the precise role of these various particles in the infectious cycle and immune modulation is not well understood we sought to determine whether virions grown in the presence of the SCD1 inhibitor had uncleaved prM protein similar to immature virus. We purified viruses from Huh7 cells infected with DENV2 at an MOI = 3 under four conditions: untreated (WT), immature virus (treated with 20mM NH_4_Cl), and virus from cells treated with the SCD1 inhibitor or with vehicle (DMSO). Virus from DMSO or SCD1 inhibitor-treated cells at 24hr post-infection were pelleted through a sucrose cushion and purified by sedimentation velocity in a potassium tartrate step gradient. Visible bands (S6D Fig) were analyzed for viral RNA and infectious virus. A majority of the viral RNA and infectivity from both treatments sedimented in fractions 5–7 (S6E and S6F Fig). To characterize the physical properties of the virus particles in each gradient we collected and similarly processed cell culture supernatants from all four conditions at 72hr post-infection, a time-point with sufficient virus for purification and analysis. The bands observed in gradients for all samples were similar to those obtained from the gradient analysis of virus harvested at 24hr with the exception of the top band (fraction 2 at the top of the 10% interface) that was more prominent after 72hr, indicating the presence of low molecular weight material (S6G Fig). We collected each band separately and characterized them for viral RNA (S6H Fig), prM, capsid and envelope proteins (Figs 7 and S6I). Quantification of the western blots is shown in Fig 7C–7F. The WT virus primarily sedimented at the 15–20% interface (fraction 6) and the 20–25% interface (fraction 8). Both of these fractions had similar levels of viral RNA. Typically, DENV2 purified from mosquito cells sediments in the 20–25% fraction, which was the primary fraction previously used for structure elucidation [30]. DENV2 produced in Huh7 cells show differences in sedimentation profiles. Immature virus sedimented at the same densities as WT virus (fractions 6 and 8) and showed the expected enrichment in prM protein compared to envelope protein (Fig 7C and 7D, fraction 6), but had less capsid protein compared to the WT virus. The virus isolated from SCD1 inhibitor-treated cells showed similar patterns of enrichment of the prM protein as the immature virus, and this enrichment was confined to the virus population in fraction 6 (S6G and S6I Fig). The virus population that sedimented in fraction 8 had no detectable prM. The virus isolated from vehicle-treated (DMSO) cells that sedimented in fraction 6 had a similar protein content to WT virus, but there was a prominent population that sedimented in fraction 4 that did not have high GE (S6H Fig). Based on these analyses, the fractions with the highest GE (fractions 6 and 8) demonstrated the clearest differences between the four conditions. The virus isolated from SCD1 inhibitor-treated cells was similar to immature virus in prM content and was distinctly different from WT virus.

## Discussion

Previously we showed that lipid biosynthesis was upregulated in DENV-infected cells through the activation and relocalization of fatty acid synthase (FAS), an enzyme critical to the production of palmitic and stearic acids that are structural components of complex lipids [14]. Here, we investigated the next step following FAS-catalyzed fatty acid production in the lipid biosynthesis pathway and demonstrated that desaturation of these fatty acids plays a critical role in the viral life cycle. Specifically, DENV2 infection resulted in upregulated monounsaturated fatty acid (MUFA) biosynthesis, catalyzed by SCD1 at early time points post-infection. Inhibition of this process impaired virion maturation and particle infectivity and stability.

Two distinct scenarios were observed (quantitatively and visually) in this study: early during infection when low levels of viral RNA and protein were present, SCD1 transcripts, protein and enzymatic activity levels were elevated. However, late during infection, when high concentrations of viral RNA were present, SCD1 mRNA, protein and activity levels declined. As summarized in Fig 7F, we hypothesize that the metabolic environment required to progress from early to advanced infections changes and SCD1 activity could function as an enzyme that modulates these changes. For instance, at early stages of viral RNA replication, increased amount of fatty acids, specifically MUFAs, are generated (through activity of SCD1) for construction of virus replication compartments. The unique architecture of positive-strand RNA virus replication compartments likely requires certain lipid components that can induce membrane curvature to provide extensive membrane contact sites and increased fluidity and leakiness to acquire substrates for genome replication [8, 16, 17, 18]. Energy and lipid substrates are provided by increased glycolysis and activation of the pentose-phosphate pathway [31]. Increased SCD1 activity also results in the build-up of storage lipids, which reduces levels of β-oxidation. The high content of MUFAs in the membranes (resulting from SCD1 activity) also ensures appropriate assembly and maturation of the virus particles being released during early infection. However, during advanced infections, when viral RNA replication is at maximum efficiency, the cell has excess complex fatty acids that feed back to inhibit SCD1 expression [32]. By the time this occurs, the virus has already constructed its replication compartments and sites of assembly and does not require synthesis of new components by SCD1. At this later time point, the focus is on producing a massive explosion of virus particles from the already assembled viral replication factories. This massive output of virus compromises quality control and increases the probability of producing mixed populations (structurally diverse) of virus particles. In these advanced infections, the decrease in MUFAs through the inhibition of SCD1 negatively influences virus particle quality, resulting in the production of higher ratios of non-infectious particles.

Viral replication compartments are tightly coordinated with sites of virion assembly. For flaviviruses, only replicated viral RNA is packaged into newly assembled virions [33]. Therefore, the lipid membrane environment surrounding viral RNA replication complexes and that involved in virion assembly must be physically coordinated to transfer newly replicated viral genomes to sites of virion assembly [8]. Essentially, any lipid alterations that occur in these complex, virus-induced membranes must retain the capacity to support multiple functions. This is especially important since intracellular membranes become structural components of virus particles (virion envelope) that require assembly of a specific stoichiometry of viral glycoproteins. Conformational transitions that occur between these glycoproteins in lipid membranes define the state of maturation and infectivity of virus particles. Accordingly, we found that virion infectivity decreased when SCD1 was inhibited early in infection. At the given dose of the SCD1 inhibitor, DENV2 was able to replicate and produce particles with intact genomes, however a higher proportion of these particles were non-infectious. We and others have provided evidence that SCD1 inhibition results in lowering MUFAs in cellular membranes. Our observations suggest that the physical properties of the virus envelope influenced by the proportion of MUFAs may be critical for virus infectivity.

DENV2 populations grown in the presence of the SCD1 inhibitor were found to be as stable to increased temperature as control virus, however, when the virus was subjected to multiple freeze-thaw cycles, it lost its infectivity faster than untreated virus. Inhibition of SCD1 results in changes in lipid content of the ER, resulting in an increase in saturated fatty acids [34, 35]. At lower temperatures, saturated fatty acids pack together tightly, forming a rigid membrane [36, 37, 38]. Rigid membranes are not able to curve well and this may impair interactions with the trans-membrane glycoproteins [36, 38]. Hence we observed a greater loss of functionality or infectivity of viral particles from SCD1-inhibited cells when transitioning from freezing to ambient temperature versus transitioning from higher temperatures, where lipids may achieve greater fluidity [39].

The defect in infectivity of virus particles from SCD1-inhibited cells apparently resulted from incomplete particle maturation. After acquiring its ER-derived lipid envelope with inserted, stoichiometrically assembled prM and E glycoprotein heterodimers, the newly formed virions traverse the Golgi apparatus to complete a maturation process prior to exiting the cell. Maturation includes pH-dependent conformational transitions between the prM and E glycoproteins embedded in the envelope that are pre-requisite for the cleavage of the prM protein to M protein in mature virions by a Golgi-resident furin protease. If the conformational changes are inhibited or incomplete, furin cannot access the cleavage site on prM to complete the maturation process. This results in prM retention on the virions; these virions are non-infectious. Based on our observations in this study, the virus requires MUFAs in its lipid envelope to allow the necessary structural transitions for virion maturation. SCD1 inhibition results in decreased proportions of MUFAs in intracellular membranes destined to become virion envelopes, resulting in impaired conformational shifts necessary for maturation and increased release of prM containing virions. We demonstrated that virions with higher prM content were defective in viral entry in subsequent infections. This establishes for the first time that lipids incorporated into the virion envelope are critical for particle infectivity.

Enveloped viruses must acquire their lipid envelope from a specific organelle membrane [40, 41, 42, 43]. Our data suggest that DENV2 assembly may occur preferentially and successfully at ER-membrane regions with a high content of MUFAs. Future research will explore the content of specific lipid species at sites of viral assembly and the consequences of their alterations. The lipid composition of the DENV2 virion is currently undetermined, thus the content of MUFAs in the infectious virion envelope is unknown. Studies of the lipid composition of other enveloped viruses have focused on lipid classes such as phospholipids and sphingolipids but have not looked at fatty acid content or saturation levels. However, it is clear that certain lipid species are enriched in viral envelopes and are functionally relevant for virion infectivity [44, 45, 46, 47, 48].

This study provides insights on how fatty acid biosynthesis, specifically unsaturated fatty acids impact flavivirus genome replication and assembly of infectious viral particles in human cells. It sheds light on host metabolic pathways that enhance viral replication success and provides an unique avenue for antiviral intervention.

## Materials and methods

### Cells lines

The cell lines used were as follows: Human embryonic Lung epithelial cells (HEL 299) (ATCC CCL-137, male), adenocarcinomic human alveolar basal epithelial cells (A549) (ATCC CRM-CCL-185, male), C636 (ATCC CRL-1660, larva, sex unknown), Clone 15 (ATCC CCL-10) of the Baby Hampster Kidney Clone 21 cells (BHK-21), and Human hepatoma (Huh7) (From Dr. Charles Rice, sex unknown, [49]. Huh7, HEL and A549 cells were maintained in Dulbeccos Modified Eagle Medium (DMEM) (Gibco, LifeTech), while BHK and C636 were maintained in Minimum Essential Media (MEM) (Gibco, LifeTech), both supplemented with 0.1 mM nonessential amino acids, and 0.1 mM L- glutamine, and 10% Fetal Bovine Serum (Atlas Biologicals) at 37°C with 5% CO_2_.

### Viruses

The virus strains used are as follows: DENV1 (16007) [50, 51], DENV2 (16681) [50, 52], DENV3 (16562) [53, 54, 55], DENV4 (1036) [53], YFV 17D [56], and KUNV [57] these viruses were passaged in C6/36 cells. Additionally, ZIKA (PRVABC59) [58] was passaged in African Green Monkey Kidney Epithilial cells from the Vero lineage (Vero) (ATCC CRL-1586). A DENV luciferase reporter replicon containing only the nonstructural proteins was also used [14]. Virus titers were determined by plaque assay on BHK cells as described previously [59]. Infection of cells was carried out at room temperature for one hour to allow virus to adhere to cells. Virus was then removed, cells rinsed with 1XPBS, overlaid with the indicated media and transferred to the 37°C incubator for required periods of time.

### RNA extraction and qRT-PCR

RNA was extracted from cells using Trizol (ThermoFisher) and from virus in supernatant using Trizol LS (ThermoFisher). A one-step qRT-PCR kit with SYBR green from Agilent was used. Reactions were set up according to the manufacturer’s protocol and run on a LightCycler 96 real-time PCR machine (Roche). The cycling parameters were: 20 mins at 50°C for reverse transcription, then 5 mins at 95°C followed by 45 two-step cycles of 95°C for 5 seconds and 60°C for 60 seconds. This was followed by a melt curve starting at 65°C and ending at 97°C. DENV primers [60] were used to quantify viral RNA copies in the supernatant as well as in cells. A standard curve of *in vitro* transcribed viral RNA from a DENV2 cDNA subclone was generated and used to quantify the genome copies in the supernatant [61]. Copies of viral RNA in the cell as well as copies of SCD mRNA transcripts were both normalized to glyceraldehyde 3-phosphate dehydrogenase (GAPDH) RNA using the delta delta ct method [62]. For this method: the fold change in gene expression = 2^(-(Infected samples((Ct value of gene of interest)–(Ct value of control gene)))–(Uninfected samples ((Ct value of gene of interest)–(Ct value of control gene)))). The Ct values were generated from the Light Cycler software and the gene of interest was either SCD1 or DENV and the control gene was GAPDH.

### siRNA treatments and confirmation

Cells were transfected with pooled siRNAs (S1 Table, Fig 1A) or single siRNAs (S2 Table, Fig 1B and 1C) using RNAiMax (Invitrogen) similar to previous experiments [14] and allowed to incubate for 48hr. Cells were then infected with DENV2, or collected for knockdown confirmation or cytotoxicity tests (described below). Virus was collected and titrated with plaque assays. To confirm knockdown of mRNA transcripts RNA was extracted and qRT-PCR performed to measure SCD1 levels relative to GAPDH in SCD1 siRNA treated samples and compared to IRR treated samples using the delta delta ct method [62] described above. To confirm knockdown of protein levels cellular protein was collected in radioimmunoprecipitation assay buffer (RIPA) [150 mM sodium chloride, 1.0% NP-40, 0.5% sodium deoxycholate, 0.1% SDS (sodium dodecyl sulfate), 50 mM Tris, pH 8.0]. and separated by electrophoresis on an SDS-Page gel. They were then transferred to a nitrocellulose membrane (Bio-Rad), blocked in 5% milk and probed with SCD N-20 (Santa Cruz Biotechnology) and β-actin (Cell Signaling Technology). Secondary antibodies were IRDye 680RD and IRDye 800CW (Li-Cor). The blot was imaged on an Odyssey IR Imaging system (Li-Cor) and quantification of the signals measured with Image Studio 5.2 (Li-Cor).

### Inhibitors and fatty acid treatments

The inhibitors used were A939572 (the SCD inhibitor, MedChem Express), C75 (Cayman Chemicals) and Lovastatin (Sigma-Aldrich). Each was diluted in DMSO, added to DMEM and filtered through a 0.2μM filter before being added to cells. Inhibitors were added to cells following virus attachment. Oleic acid was acquired from Sigma and came dissolved in bovine serum albumin (BSA) at 200mM. It was further diluted in 1% fatty acid free BSA (Gold Biotechnology) in 1x phosphate buffered saline (PBS) to 50 μM. Huh7 cells were infected with virus as described above and overlaid with the indicated treatments diluted in DMEM. Supernatants were collected at 24hr and plaque assays performed. Cytotoxicity was measured with alamar blue (ThermoFisher) diluted 1:10 in DMEM incubated on cells for 2–4 hr and read on a Victor 1420 Multilabel plate reader (Perkin Elmer) with excitation at 560 nM and emission at 590 nM.

### Re-infection experiments

Virus that was used for re-infection and entry assays was grown in Huh7 cells at an MOI of 3 in 10 μM SCD1 inhibitor or DMSO (0.02%). The virus samples were titrated by plaque assay and diluted to equivalent titers. The SCD1 inhibitor was added to the DMSO sample and the virus samples (equivalent pfu/ml) were allowed to attach to new cells for 1 hr at room temperature before being removed. The cells were then washed with 1xPBS and incubated with DMEM + 2% FBS for the indicated times at 37°C. For siRNA confirmation of these results, virus was grown with siRNA treatment as described above. Virus was collected at 48hr, titrated and equal PFU were used to infect new Huh7 cells at MOI = 0.3 and overlaid with DMEM + 2% FBS. After 48hr of replication virus supernatant was collected and titrated.

### Assay for viral entry

Each virus was diluted to 1000 pfu/ml in DMEM. The SCD inhibitor was added to the virus that was grown in DMSO (0.02%) at 10 μM and then filtered. Virus was allowed to attach to BHK cells in 6 well plates for 2 hr at 4°C. The virus was then aspirated and cells were rinsed with 1XPBS to remove any unbound virus. The cells were then overlaid with MEM with 10% FBS and transferred to 37°C. At given time points after the temperature shift, cells were removed from the incubator rinsed with 1XPBS and treated with acid-glycine (8 g of NaCl, 0.38 g of KCl, 0.1 g of MgCl2 6H2O, 0.1 g of CaCl2 2H2O, and 7.5g of glycine/L, pH adjusted to 3 with HCl) for 1 minute at room temperature to inactivate any extracellular virus. The cells were again rinsed with 1XPBS and overlaid with 1% agarose and MEM with 5% FBS, plaques were counted at 6 days.

### Immunofluorescence assay

Cells were grown on a sterilized cover slip and maintained in 10% DMEM. Cells were infected with DENV2 (M0I = 100) or mock infected (1XPBS). Cells were fixed in ice-cold methanol (Fisher Chemical) at room temperature and permeabilized with 0.1% TritonX (Fisher Chemical) in 1XPBS with 1% BSA (GoldBiotech) at room temperature and blocked with 0.01% TritonX in 1XPBS with 1% BSA overnight at 4°C. Cells were then probed with the indicated primary antibodies including dsRNA (English and Scientific Consulting Bt.), NS3 (gifted by Richard Kuhn, Purdue University) and SCD N-20 (SantaCruz Biotechnology). Secondary antibodies were Alexa-fluor 488 or 647. The coverslips were fixed to slides with FluoroSave (Calbiochem) and imaged on an Olympus inverted IX81 FV1000 (Olympus) confocal laser scanning microscope with a 100x oil objective using FV10-ASW 4.2 (Olympus). Digital images were processed with Volocity 6.3 (Perkin Elmer) and colocalization coefficients calculated by encircling each individual cell and using the measurement function with internal thresholds. Values represent averages of at least 30 cells from different image frames of the same slide.

### Time of addition

Huh7 cells were infected with DENV2 (MOI = 0.5) and overlaid with DMEM. At the indicated time-point the inhibitor (10μM) or vehicle were added to cells. Virus supernatants were collected after 48hr and titrated. To look at the pre-infection and attachment phases, we added the inhibitor to Huh7 cells and then infected cells after 12hr of treatment. After infection we washed cells and overlaid with either the inhibitor, vehicle control or DMEM. We also added the inhibitor or vehicle control to the virus inoculum, allowed attachment to occur for 1 hr, then washed cells and either added new inhibitor or removed it to determine an impact on the attachment stage.

To confirm findings with the siRNA we infected Huh7 cells with DENV2 (MOI = 0.1) and overlaid with DMEM + 2% FBS. After 24hr of replication, the cells were transfected with the indicated siRNAs. Viral supernatant was then collected and titrated at 48 and 72hr post infection.

### SCD1 activity assay

The indicated cell samples were collected at given time points and prepared in order to preserve enzymatic activity [63]. Briefly, cells were washed in the wash buffer (35 mM Hepes, pH 7.4, 146 mM NaCl, 11 mM glucose) 3 times, then incubated in a hypotonic solution (20 mM Hepes pH 7.4, 10 mM KCl, 1.5 mM MgOAc, 1mM DTT) for 20 minutes to allow the cells to swell. Then they were passed through the dounce homogenizer 25 times to break apart the membranes. A post lysis buffer (20 mM Hepes pH 7.4, 120 mM KOAc, 4mM MgOAc, 5 mM DTT) was added. Nuclei were spun down at 1000xg for 5 minutes at 4°C and the cytoplasmic extract was flash frozen in liquid N_2_. Protein content was measured by BCA (Pierce) and equal protein content was used for activity assays. Activity assays were performed similar to previous studies [64]. Briefly, 1.5 mg/ml protein was incubated with 0.01 μCi of stearoyl [1-14C]-coA (American Radiolabeled Chemicals, 55mCi/mM) at 37°C for 5 minutes. The reactions were stopped by adding 150 μL of methanolic HCl (Sigma-Aldrich) for 1 hour at 72°C, which generated fatty acid methyl esters that were extracted from the samples in 1 mL of chloroform. One aliquot of these samples was measured on a Beckman LS 6500 liquid scintillation counter (Beckman) and equal counts were spotted on a 0.5% AgNO_3_ impregnated normal phase thin layer chromatography plate. Fatty acids were separated by a mobile phase of hexane: acetone (50:1). Plates were exposed on a phosphoimager screen and scanned on a Typhoon Trio 9400 (GE Healthcare). Pixel intensity was measured with ImageQuant TL(GE Health Care) software. Standards were sprayed with 5% phosphomolibic acid in 100% ethanol and heated to visualize fatty acids.

### LC/MS and data analysis

Huh7 cells were infected with DENV2 (MOI = 10), a UV-inactivated virus or mock-infected. Cells were collected at the indicated time points and metabolites were extracted from an equal number of cells per sample. A mixture of 2∶1 chloroform∶methanol, 0.1% acetic acid and 0.01% butylated hydroxy toluene (BHT) were added to the cell suspension in ammonium bicarbonate to generate a 4∶1 ratio of organic solvent to cells. The non-polar phase was removed, dried down under N_2_ stream resuspended in 75 μl of ice-cold methanol and vortexed for 10 s. The samples were then centrifuged at 13,400× g for 5 min to remove any particulates.

The samples were run on a LTQ Orbitrap XL instrument (Thermo Scientific, Waltham, MA). It was coupled to an Agilent 1100 series LC (Agilent Technologies, Santa Clara, CA). An Xterra C18 column (Waters Corp., Milford, MA) was used in reverse phase. Solvent A consisted of water + 10mM ammonium acetate + 0.1% formic acid. Solvent B was acetonitrile/isopropyl alcohol (50/ 50 v/v) + 10mM ammonium acetate + 0.1% formic acid. The flow rate was 300 μL/minute. A sample volume of 10 μL was loaded onto the column. The gradient was as follows: time 0 minutes, 35% B; time 10 minutes, 80% B; time 20 minutes, 100% B; time 32 minutes, 100% B; time 35 minutes, 35% B; time 40 minutes 35% B. The LC-MS analysis was run twice, with negative polarity ESI. The acquired data were evaluated with Thermo XCalibur software (version 2.1.0).

The raw data was converted to mzXML format with msConvert(46). Peak picking was accomplished with the XCMS package using centWave(47–49) and the IPO package to optimize parameters(51). Intensities for peaks were determined and normalized using the median fold change method (52,53). Features were annotated with the *mummichog* software version 2.0(21).

### Virus purification

Virus purification and identification of prM were carried out similar to previous studies [30, 65]. Briefly, Huh7 cells were infected with DENV (MOI = 3) and left untreated, treated with 10 μM of the SCD inhibitor or DMSO similar to other experiments presented here. The supernatant was collected at the indicated time points and was replaced with fresh DMEM plus the indicated treatment. At 24hr after infection 20mM NH_4_Cl was added to generate the immature virus samples. The supernatants from the SCD inhibitor and DMSO samples were collected at 24hr. Cellular debris was removed from the supernatant and the virus was run through a 22% sucrose cushion at 32,000 xg for 2 hr at 4°C in a Sorvell WX ultracentrifuge (ThermoFisher Scientific). The pellet was loaded onto a potassium-tartrate step gradient consisting of 10, 15, 20, 25, 30 and 35% potassium-tartrate in TNE buffer (50 mM Tris–HCl (pH 7.4), 100 mM NaCl, 0.1 mM EDTA) and spun at 32,000 xg for 2 hr at 4°C in a Sorvell WX ultracentrifuge (ThermoFisher Scientific). Ten different fractions were collected from each gradient and used to titrate the virus and quantify viral genomes similar to other experiments. At 72 hr virus from all 4 samples were collected, cellular debris removed and virus was PEG precipitated at 4°C. The virus was then run through a 22% sucrose cushion and loaded onto a potassium-tartrate gradient same as mentioned above. Visible band of virus were noted in the gradients and the 4 fractions were collected and buffer exchanged with TNE buffer. Viral genome copies were measured with qRT-PCR. Protein content was measure with BCA (Pierce/ThermoFisher) and equal amounts of protein were loaded on an SDS-page gel and transferred to a PVDF membrane and probed for prM, capsid and E. Blots were imaged on an Odyssey and densitometric measurements were made. Western blots for each fraction (i.e.: fraction 6) had equal total protein loaded for the four conditions. Conditions between fractions cannot be compared due to differences in protein sedimentation between fractions (i.e.: total protein in fraction 6 cannot be compared to total protein in fraction 8).

### Quantification and statistical analysis

Results were expressed as mean values with standard deviation. The statistical details are noted in the figures and/ or in the corresponding figure legends. Statistical significance was primarily determined using either an unpaired Student's t-test or a one or two way Analysis of Variance (ANOVA) with a Bonferroni, Dunnets or Tukey’s multiple-comparison depending on the experimental design in the GraphPad Prism version 7.00 for Mac OS x (GraphPad Software, La Jolla California USA). Drug inhibition studies were analyzed with a non-linear regression using GraphPad Prism version 7.00 for Mac OS x (GraphPad Software, La Jolla California USA) to calculate an IC50. The assay for viral release (Fig 5A) was analyzed with a 3-way ANOVA and included an interaction term for each. This was performed in R studio version 1.0.136 [66]. Finally, the nonlinear regression models for the thermostability of the viruses (S6A Fig) was also performed in R studio version 1.0.136 [66]. The model used was y = a+((b-a)/((1+10^((c-temp)*d))). An F-test was used to determine the difference between the curves with the formula F = ((SS_total_-SS _pooled_) / ((m+1)(K-1))) / (SS_pooled_/df_pooled_). Each study shown is representative of at least two independent experiments.

Additional key resources are included in S2 Table.

## Supporting information

S1 Table Related to Fig 1. **siRNA screen of the human unsaturated fatty acid biosynthesis pathway.** The enzymes in the UFA biosynthesis pathway were identified based on the KEGG database [67]. Here each enzyme is listed along with its gene ID, Accession number, GI number (GenInfo Identifier) and the sequence for the four siRNAs used to knockdown each gene.
(PDF)

S2 Table Key resources table. Here we have information regarding reagents and resources used for this manuscript.
(PDF)

S1 Fig Related to Fig 1. **siRNA screen of the human UFA biosynthesis pathway (cytotoxicity and validation).** Multiple cell types were transfected with single siRNAs targeting enzyme “hits” initially identified in the pooled siRNA screen of the pathway. Infectious virus release from siRNA treated cells and controls was measured. (A) Huh7 cells, (B) A549 cells. A one-way ANOVA with multiple comparisons was done. (C) Cytotoxicity of the siRNAs (in Fig 1) was measured by the fluorescence of the reduction of resazurin to resorufin. A one-way ANOVA with multiple comparisons was done; none of the treatments were significantly cytotoxic. Cytotoxicity of the single siRNAs in Huh7 cells are shown in (D) without virus addition and (E) with virus addition. (F) qRT-PCR analysis to confirm knockdown of SCD1 gene expression. (G) Western blot analysis to confirm knockdown of SCD1 protein using antibodies against SCD1 and Actin. Signal intensities are quantified One-way ANOVA indicated no significant difference. (ns = not significant, * = p = 0.05, ** = p<0.001, **** = p<0.0001 compared to IRR)
(TIF)

S2 Fig Related to Fig 2. **NS3 co-localizes with SCD1 in certain cell types. A.** Huh7 cells were mock infected and fixed in ice-cold methanol at the indicated time points. Cells were permeabilized and probed with the indicated antibodies. (B). Huh7 cells on cover slips were transfected with an irrelevant (IRR) siRNA or one specific for SCD1 and fixed after 48hr to ensure complete degradation of SCD1 mRNAs and turnover of the SCD1 protein. Cells were then permeabilized and probed for SCD1 with an Alexafluor 647 secondary antibody. The 647 signal is shown in the top two panels with DAPI in the bottom panels. (C). The signals from these cells were quantified and we see less 647 signal in cells treated with the SCD1 siRNA. An unpaired t-test showed a significant difference with p<0.05. (D) and (E). Human embryonic lung (HEL) cells and A549 cells were infected with DENV for 36 and 24hr respectively and processed similarly to A. Inset shows a 3-D reconstruction of a infected A549 cell. (F). Quantification of signals and co-localization coefficients of A549 cells. In both cell types uninfected cells show expression of SCD1, but infected cells show accumulation at perinuclear sites. (* = p<0.05)
(TIF)

S3 Fig Related to Fig 3. **Inhibition of SCD1 in other cell types.** A dose response curve of SCD1 inhibition of DENV2 replication in C6/36 cells (A) and A549 (B). Cells were infected with DENV2 (MOI = 0.5) and treated with the indicated concentrations of the SCD1 inhibitor. Virus supernatant was collected at 24hr post infection and quantified by plaque assay. Cytotoxicity was measured by the fluorescence of the reduction of resazurin to resorufin.
(TIF)

S4 Fig Related to Figs 3 and 4. **Time of addition of SCD1 inhibitor and siRNA.** (A-C) Huh7 cells were infected with DENV2 (MOI = 0.5), overlaid with DMEM, the inhibitor was added at the indicated time points, and virus supernatant was collected at 48hr. (A) The inhibitor was added to cells at 12hr prior to infection and then either removed or kept on for 48hr, or the inhibitor was added after adsorption of the virus (time = 0). (B) The inhibitor was added during the attachment stage and then either removed or retained for 48hr, or the inhibitor was added after adsorption of the virus (time = 0). (C) The inhibitor was added at the indicated timepoints and virus supernatants were collected at 48hr. (D-E) Huh7 cells were infected with DENV2 (MOI = 0.1) and incubated for 24hr. Then the indicated siRNAs were added to the cells. Supernatant was collected and titrated at (D) 48 and (E) 72hr post infection. (ns = not significant, * = p<0.05, ** = p<0.005, *** = p<0.0005, **** = p<0.0001 compared to control, ^#^These virus samples are the same and is shown twice for comparison to the other data).
(TIF)

S5 Fig Related to Figs 5 and 6. **DENV2 treated with SCD1 inhibitor has a defect in infectivity in human cells but not mosquito cells.** (A) Huh7 cells were infected with ZIKV and treated with the SCD1 inhibitor for 24hr. Supernatant was collected and quantified by plaque assay. RNA was extracted and genome copies measured by qRT-PCR. (B) C636 cells were infected with DENV2 and treated with the SCD1 inhibitor for 24hr. Supernatant was collected and quantified by plaque assay. RNA was extracted and genome copies measured by qRT-PCR. (C-F) Huh7 cells were infected with DENV2 (MOI = 0.5) and treated with C75 or DMSO (C, E) or Lovastatin or DMSO (D, F). Supernatants were collected at the indicated time points and viral titer determined by plaque assay (C, D) or RNA was extracted and genome equivalents measured by qRT-PCR (E, F). (G-H) Cells were infected with DENV2 (MOI = 3) and treated with 10μM SCD1 inhibitor. This virus was collected at 24hr and subsequently used to re-infect new cells at MOI = 0.1 in the absence of inhibitor. Supernatant was collected at 24hr and viral titer determined by plaque assay. (G). Experiments in Huh7 cells. (H). Experiments in A549 cells. (ns = not significant, * = p<0.05, ** = p<0.005, *** = p<0.0005, **** = p<0.0001 compared to control)
(TIF)

S6 Fig Related to Fig 7. **Characterization of virus grown in the presence of SCD1 inhibitor.** Huh7 cells were infected with DENV2 and treated with the SCD1 inhibitor or vehicle. Supernatant was collected at 24hr and the viral titer quantified by plaque assay. (A). The virus was then diluted to 1000 pfu/ml and subjected to the indicated temperature for 15 minutes. The virus was allowed to recover at room temperature and then used to infect BHK cells. Plaques were counted and the PFU/mL was calculated. (B-C). The indicated virus samples were subjected to freeze/thaw cycles. Virus was thawed, titrated on BHK cells and returned to -80°C until frozen. (B) DENV2 was cycled through this process 5 times. A linear regression was performed and the control samples yielded a slope of 0.06 that was not significantly different from zero, while the SCD1 inhibitor samples yielded a slop of -0.75 that significantly deviated from zero with p = 0.02. (C) ZIKV was cycled through this process 4 times. (D-I) Huh7 cells were infected with DENV2 and left untreated (WT), treated with 20mM NH4Cl (immature), 10mM SCD1 inhibitor or vehicle (DMSO). (D-F) Virus grown with the SCD1 inhibitor or DMSO was collected at 24hr, concentrated through a sucrose cushion and run on a K-tartrate gradient. (D) Ten fractions were collected (labeled 1–10). Distinct bands observed are shown in grey. The virus in each fraction from DMSO (E) or SCD1 inhibitor treated samples (F) was titrated (black) and RNA from each fraction was extracted to measure genome equivalents (grey). (G-I) Virus supernatant was also collected at 72hr post-infection, PEG precipitated, concentrated through a sucrose cushion, and purified on a K-tartrate gradient. (G) Distinct bands (fractions 2, 4, 6 and 8) where virus was observed (grey) were concentrated, and buffer exchanged. (H) RNA was extracted from these bands to measure genome equivalents and (I) Western blots performed to probe for envelope, capsid and prM viral proteins. *These data (for fractions 6 and 8) were also shown in Fig 7.
(TIF)
