# Supplementary material for: Stearoly-CoA desaturase 1 differentiates early and advanced dengue virus infections and determines virus particle infectivity
Source: PLoS Pathog. 2018 Aug 17;14(8):e1007261. doi: 10.1371/journal.ppat.1007261 (PMC6114894; doi:10.1371/journal.ppat.1007261)
Supplement: S1 Table — siRNA screen of the human unsaturated fatty acid biosynthesis pathway. The enzymes in the UFA biosynthesis pathway were identified based on the KEGG database [67]. Here each enzyme is listed along with its gene ID, Accession number, GI number (GenInfo Identifier) and the sequence for the four siRNAs used to knockdown each gene. (PDF) [file ppat.1007261.s001.pdf]

Gene Symbol	Gene ID	Gene Accession	GI Number	Sequence 1	Sequence 2	Sequence 3	Sequence 4
ELOVL6	79071	NM_024090	13129087	CAAUGGACCUGUCAGCAAA	GGUCGGCACCUAAUGAAUA	CGAACUAGGAGAUACAAUA	GGGUGUAUAUCUAGAACGA
HSD17B12	51144	NM_016142	153792624	GAAAUCGGCAUCUUAGUGA	UAAGAUGACACAAUUGGUA	CGUAUGAGUAUCCUGAAUA	GAACUAAUAUUGUCGGGAA
PECR	55825	NM_018441	93102372	GAGGAUCUAUCGUCAAUAU	GGACCUUUCUGUUGUCAAA	AGGAGGAGGUGAAUAAUUU	GAAUGGGCCUGCAGUGGAA
PTPLB	201562	NM_198402	158819030	GAGAACUGCUCACAAUAUA	CGGCGUACCUGGUCAUCUA	AAUCAUCCGUUACUCCUUU	CAUGGACGAUCACGGAAAU
PTPLA	9200	NM_014241	82659104	UGAGAUAGUUCACUGUUUA	CAAUAAGACUUCCUAACAA	CCACAACUCUAUUUUCAUA	UGACAGAGAUCACUCGCUA
GPSN2	9524	NM_138501	50726974	UGGAGAUUCUGGACGCAAA	AUUACGAGGUGGAGAUUCU	GAGCUCAGCAGGUGAAACU	GAUUCUGGACGCAAAGACA
SCD	6319	NM_005063	53759150	GAUAUGCUGUGGUGCUUAA	AGAAUGAUGUCUAUGAAUG	CGACAUUCGCCCUGAUAUA	GGAGUACGCUAGACUUGUC
SCD5	79966	NM_024906	148596937	CAUAUUGGGUGGCUGUUUG	AGAACAUCGUCUGGAGGAA	GAGAAAGCUUGACGUCACU	CAGAAUGACAUCUUCGAGU
FADS2	9415	NM_004265	14141180	GCACUACGCUGGAGAAGAU	UGAAAUACCUGCCCUACAA	GGCAAGAACUCAAAGAUCA	GGCAAUGGCUGGAUUCCUA
ELOVL5	60481	NM_021814	52851443	CAAGGAAGCUGCGGAAGGA	GGUUUCUUCUGGACAAUUA	UCACACUGCUGUCUCUGUA	UCUCCAAACUCAUAGAAUU
FADS1	3992	NM_013402	14141179	GGAACCAUCUGCUACAUCA	GAGGAGCGGUGGCUAGUGA	GACUUGGCCUGGAUGAUUA	GCAUAGAGUACCAGUCCAA
ELOVL2	54898	NM_017770	157388944	CAAAUGGAGUGAUGAACAA	CCAGUCAUCUUAUAUGCUA	CGGUCAUGAGCCACGAUAA	CAAUAUGUUUGGACCGCGA
ACOX1	51	NM_007292	83641872	GGAAAGACUUCAAAUCAUG	GGGCAUGGCUAUUCUCAUU	CAAGUAAACCAGCGUGUAA	UUACAUGCCUUUAUCGUAC
ACOX3	8310	NM_001101667	156104865	GCAAGCGGAUCUUCGAGUA	ACAAGUGGCUGGUUUGCUA	GAUCGCUCCUCCUGACUUU	GAAUUAAGCCACGGCAGUA
HADHA	3030	NM_000182	105990523	GCUCUAACAUCAUUUGAAA	UCUCAGAAGUUAUGAAUGA	GACAAUAGAAUACCUAGAA	CGAAACAUGUGGCGGAAGA
ACAA1	30	NM_001607	6598316	GAGAUUGCCUGAUUCCUAU	GGGAUAACCUCUGAGAAUG	CCACCACGGUCCAUGAUGA	GAAAUAUUACUUCGCGCUU
ZAP128	10965	NM_006821	148727285	GGAAACAACUCCAGACUUU	GGAAGGACCUGACCAGAAG	GAGGUGGCCUGCUGGAGUA	GACCAAAGAUGGCUAUGCA
BACH	11332	NM_181866	75709215	GGCGGUACCUGCAGAUGAA	GCGCGGAGAUCACCUACAC	GCGCACCGACUUCCUGUCU	GACGAGAAGAAGCGCUUUG
PTE2B	122970	NM_152331	63999751	UAUAAGGAAUGCUCUCGUA	CCAAUAACAUGGACAACAU	CAUCGAGCCUCCUUACUUC	GAAGCGGGACGUACAGAUU
ACOT1	641371	NM_001037161	81230484	GGGACGAACCGGUGCGAAU	CUGCUGGAGUAUCGGGCUA	CCAAAGAUGGCUAUGCAGA	CAACAGAAAUCGCAUCAAG
BAAT	570	NM_001701	4502350	GGAAGGAGAUCCAGAGAUU	GACUAUCUAUGGCUAUUUA	CAAUUAAUAUCCACCAAUG	CCACGGUACUUAUUAAUGG
