# Supplementary material for: Stearoly-CoA desaturase 1 differentiates early and advanced dengue virus infections and determines virus particle infectivity
Source: PLoS Pathog. 2018 Aug 17;14(8):e1007261. doi: 10.1371/journal.ppat.1007261 (PMC6114894; doi:10.1371/journal.ppat.1007261)
Supplement: S2 Table — Here we have information regarding reagents and resources used for this manuscript. (PDF) [file ppat.1007261.s002.pdf]

REAGENT or RESOURCE	SOURCE	IDENTIFIER
**Antibodies**		
anti- SCD Antibody (N-20)	Santa Cruz Biotechnology	Cat#: sc-14715 RRID: AB_2238791
anti- β-Actin (8H10D10) Mouse mAb	Cell signalling	Cat#: 12262 RRID: AB_2566811
anti-dsRNA monoclonal antibody	English & Scientific Consulting Bt	Cat#: 10010500 RRID: AB_2651015
anti-NS3	Dr. Richard Kuhn(Heaton et al. 2010a)	NA
anti-Envelope (4G2)	Dr. Richard Kuhn(Gentry et al. 1982)	NA
anti-Dengue 2 virus PrM protein antibody	Genetex	Cat#: GTX128093 RRID: AB_1240702
anti-Dengue 2 virus Capsid protein antibody	Genetex	Cat#: GTX103343 RRID: AB_1240697
Chicken anti-Goat IgG (H+L) Secondary Antibody, Alexa Fluor® 647 conjugate	Thermo Fisher Scientific	Cat#: A-21469 RRID: AB_2535872
Donkey anti-Mouse IgG Secondary Antibody, Alexa Fluor® 488 conjugate	Thermo Fisher Scientific	Cat#: R37114 RRID: AB_2556542
IRDye® 680RD Donkey anti-Goat IgG (H + L), 0.5 mg	Li-Cor	Cat#: 926-68074 RRID: AB_10956736
IRDye 800CW Goat anti-Mouse IgG (H + L), 0.5mg	Li-Cor	Cat#: 926-32210 RRID: AB_2687825
IRDye 800CW Goat anti-Rabbit IgG (H + L), 0.5mg	Li-Cor	Cat#: 926-32211 RRID: AB_10796098
IRDye® 680RD Goat anti-Mouse IgG (H+L), 0.5 mg	Li-Cor	Cat#:926-68070 RRID: AB_2651128
**Bacterial and Virus Strains**		
DENV1 (16007)	Dr. Clair Huang, CDC (Yoksan, 1986; Huang et al. 2000)	AF180818
DENV2 (16681)	Dr. Clair Huang, CDC (Yoksan, 1986; Kinney et al. 1997)	U87411
DENV3 (16562)	Dr. Clair Huang, CDC (Jirakanjanakit et al. 1999; Angsubhakorn et al. 1994; Goh et al. 2016)	KU725665
DENV4 (1036)	Dr. Clair Huang, CDC (Jirakanjanakit et al. 1999)	U18429
YFV 17D	Dr. Charles Rice (Rice et al. 1985)	NC_002031

KUNV	Dr. Alexander Khromykh (Khromykh & Westaway 1994)	AY274505
SINV	Dr. Richard Kuhn (Strauss et al. 1984)	NC_001547
ZIKA (PRVABC59)	Dr. Aaron Brault, CDC (Lanciotti et al. 2016)	KU501215
**Chemicals, Peptides, and Recombinant Proteins**		
siRNA library	Dharmacon (This paper)	Table S1
Lipofectamine® RNAiMAX Transfection Reagent	Invitrogen	13778075
SCD siRNA: GAUAUGCUGUGGUGCUUAA[dT][dT]	Sigma	SASI_Hs01_00181371
siIRR: Custom siRNA	Dharmacon	CTM-278879
MISSION® esiRNA esiRNA human SCD1	Sigma-Aldrich	EHU108071-20UG
MISSION® esiRNA esiRNA human ELOVL2	Sigma-Aldrich	EHU033101-20UG
MISSION® esiRNA esiRNA targeting human ELOVL6	Sigma-Aldrich	EHU005171-20UG
MISSION® esiRNA esiRNA targeting human PECR	Sigma-Aldrich	EHU002471-20UG
MISSION® esiRNA esiRNA targeting mouse Acot1	Sigma-Aldrich	EMU214111-20UG
DAPI (4',6-Diamidino-2-Phenylindole, Dihydrochloride)	Invitrogen / Life Technologies	D1306
Stearoyl [1-14C] coA	American Radiolabled Chemicals	ARC 0756-10 µCi
SCD inhibitor	Medchem Express	HY-50709
C75	Cayman	10005270
Lovastatin	Sigma-Aldrich	PHR1285
Trizol	Lifetech	15596018
Trizol LS	Lifetech	10296-028
Bovine Serum Albumin (BSA), Fraction V, Fatty Acid Free for tissue culture	Gold Biotechnology	A-421-25
Oleic Acid	Sigma-Aldrich	O1008
Stearic Acid	Sigma-Aldrich	S4751
Oleic Acid-BSA	Sigma-Aldrich	O3008
**Critical Commercial Assays**		
Brilliant III Ultra-Fast SYBR qRT-PCR Master Mix	Agilent	600886
**Experimental Models: Cell Lines**		
Huh7	Dr. Charles Rice (Blight et al. 2002)	
BHK	ATCC	ATCC CCL-10
C636	ATCC	ATCC CRL-1660
A549	ATCC	ATCC CRM-CCL-185
HEL299	ATCC	ATCC CCL-137
Vero	ATCC	ATCC CRL-1586
**Oligonucleotides**		

DEN2 +strand (FWD): ACAAGTCGAACAACCTGGTCCAT	(Laue, Emmerich, & Schmitz, 1999	
DEN2 +strand (REV): GCCGCACCATTGGTCTTCTC	(Laue, Emmerich, & Schmitz, 1999	
Stearoyl-CoA desaturase 1 (FWD): TTGGGAGCCCTGTATGGGAT	This paper	
Stearoyl-CoA desaturase 1 (REV): TTTGTAAGAGCGGTGGCTCC	This paper	
GAPDH (FWD): TCCTGTTCGACAGTCAGCCG	This paper	
GAPDH (REV): AGTTAAAAGCAGCCCTGGTGA	This paper	
**Recombinant DNA**		
DENV2 luciferase replicon	Dr. Richard Kuhn (Heaton et al. 2010a)	
**Software and Algorithms**		
GraphPad Prism version 7.00 for Mac OS x	GraphPad Software, La Jolla California USA)	
R studio version 1.0.136	RStudio Team 2016	
ImageQuant TL	GE Health Care Life Sciences	
Volocity 6.3	Perkin Elmer	
FV10-ASW 4.2	Olympus	
Image Studio 5.2	Li-Cor	
LightCycler 96 SW 1.1	Roche	
