# Supplementary figures and images for: Stearoly-CoA desaturase 1 differentiates early and advanced dengue virus infections and determines virus particle infectivity
Source: PLoS Pathog. 2018 Aug 17;14(8):e1007261. doi: 10.1371/journal.ppat.1007261 (PMC6114894; doi:10.1371/journal.ppat.1007261)

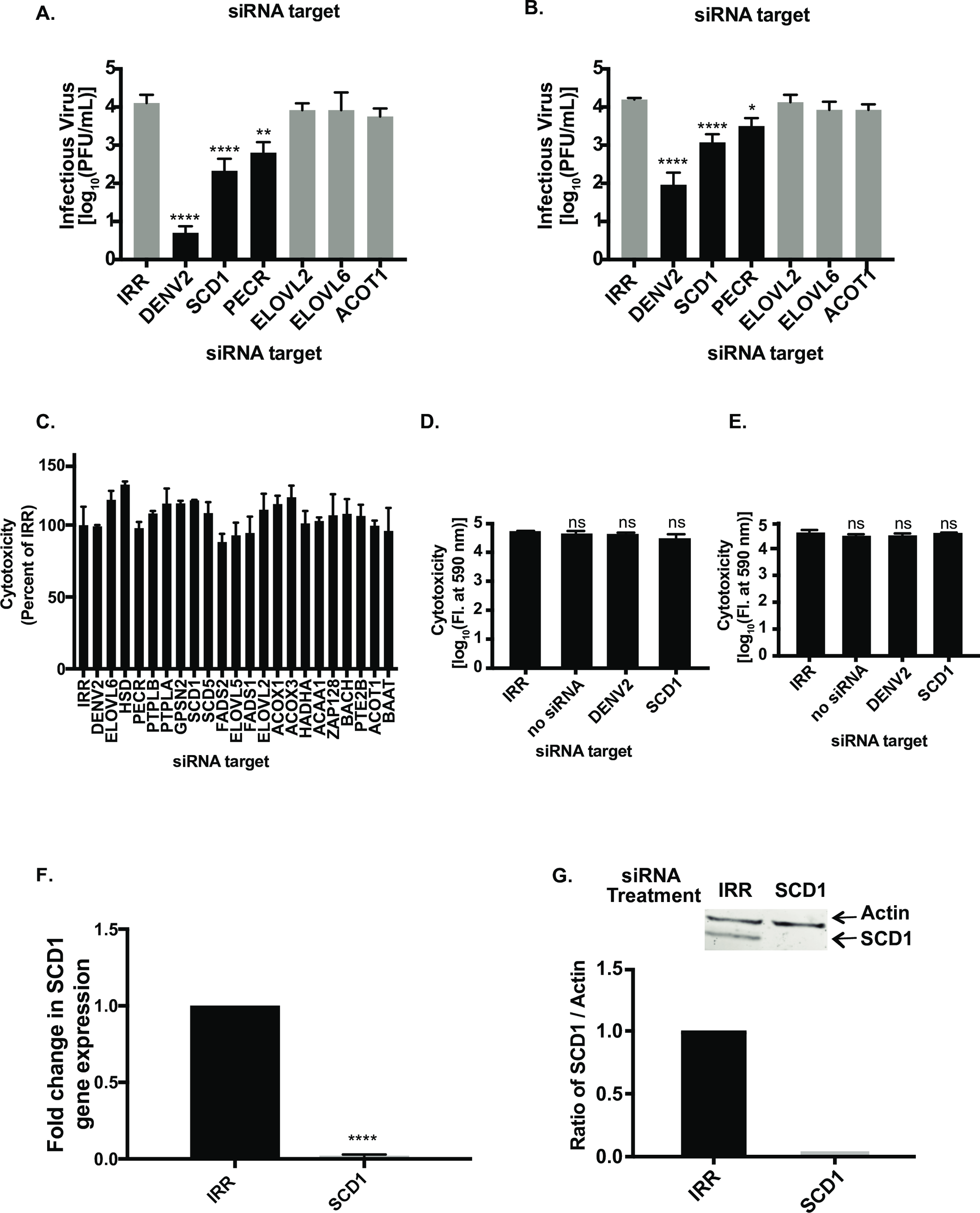

Supplement: S1 Fig — siRNA screen of the human UFA biosynthesis pathway (cytotoxicity and validation). Multiple cell types were transfected with single siRNAs targeting enzyme “hits” initially identified in the pooled siRNA screen of the pathway. Infectious virus release from siRNA treated cells and controls was measured. (A) Huh7 cells, (B) A549 cells. A one-way ANOVA with multiple comparisons was done. (C) Cytotoxicity of the siRNAs (in Fig 1) was measured by the fluorescence of the reduction of resazurin to resorufin. A one-way ANOVA with multiple comparisons was done; none of the treatments were significantly cytotoxic. Cytotoxicity of the single siRNAs in Huh7 cells are shown in (D) without virus addition and (E) with virus addition. (F) qRT-PCR analysis to confirm knockdown of SCD1 gene expression. (G) Western blot analysis to confirm knockdown of SCD1 protein using antibodies against SCD1 and Actin. Signal intensities are quantified One-way ANOVA indicated no significant difference. (ns = not significant, * = p = 0.05, ** = p<0.001, **** = p<0.0001 compared to IRR) (TIF) [file ppat.1007261.s003.tif]

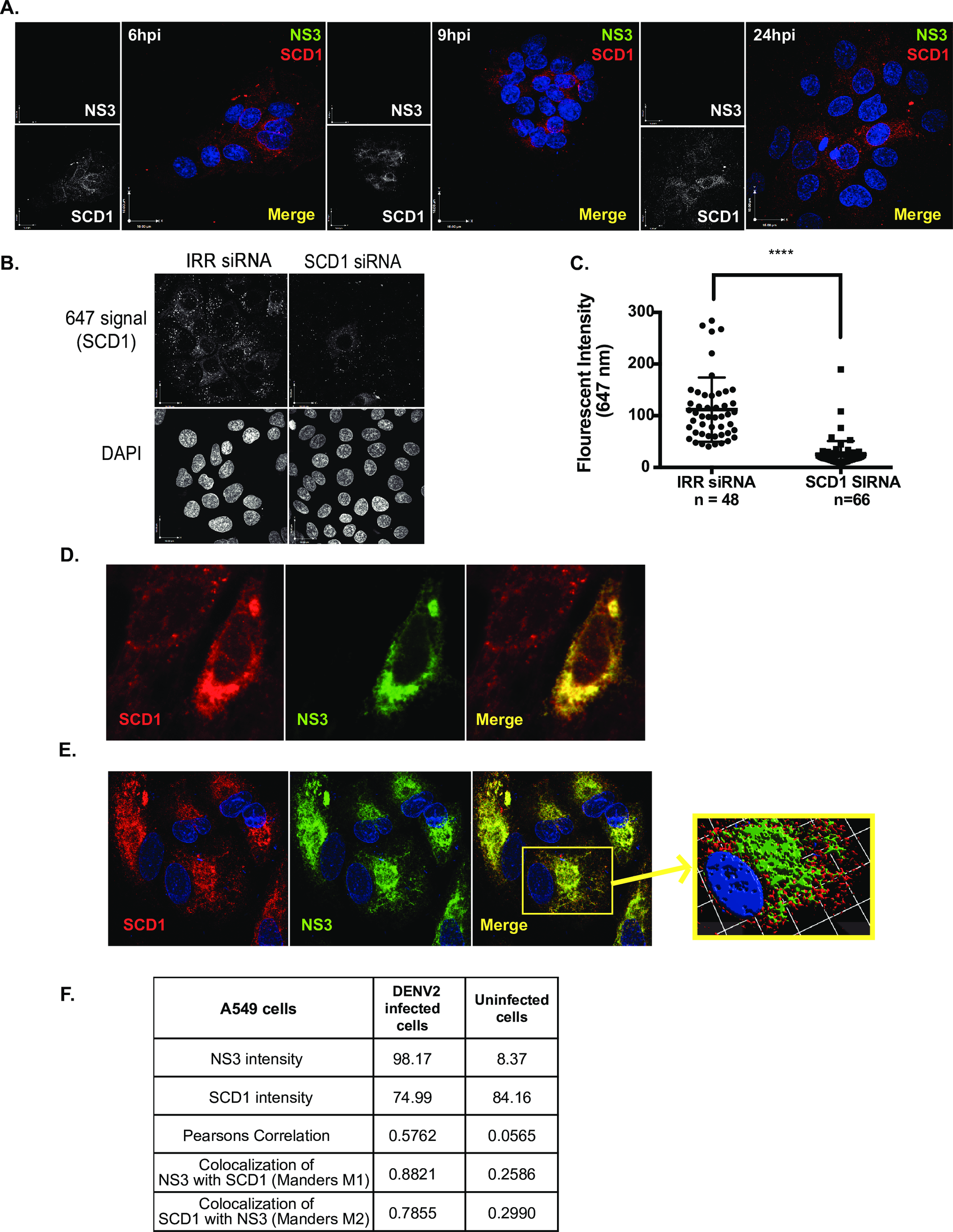

Supplement: S2 Fig — NS3 co-localizes with SCD1 in certain cell types. A. Huh7 cells were mock infected and fixed in ice-cold methanol at the indicated time points. Cells were permeabilized and probed with the indicated antibodies. (B). Huh7 cells on cover slips were transfected with an irrelevant (IRR) siRNA or one specific for SCD1 and fixed after 48hr to ensure complete degradation of SCD1 mRNAs and turnover of the SCD1 protein. Cells were then permeabilized and probed for SCD1 with an Alexafluor 647 secondary antibody. The 647 signal is shown in the top two panels with DAPI in the bottom panels. (C). The signals from these cells were quantified and we see less 647 signal in cells treated with the SCD1 siRNA. An unpaired t-test showed a significant difference with p<0.05. (D) and (E). Human embryonic lung (HEL) cells and A549 cells were infected with DENV for 36 and 24hr respectively and processed similarly to A. Inset shows a 3-D reconstruction of a infected A549 cell. (F). Quantification of signals and co-localization coefficients of A549 cells. In both cell types uninfected cells show expression of SCD1, but infected cells show accumulation at perinuclear sites. (* = p<0.05) (TIF) [file ppat.1007261.s004.tif]

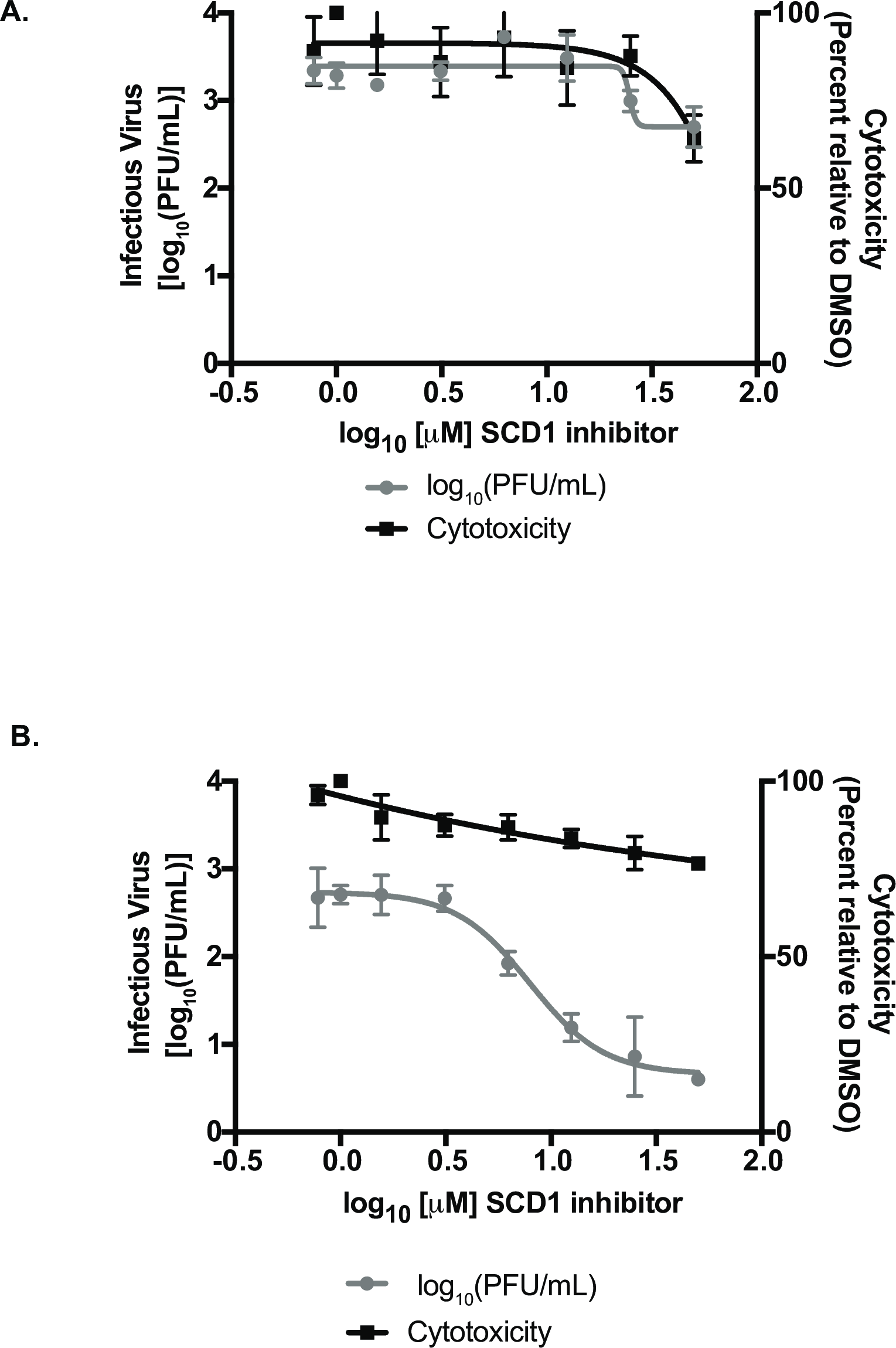

Supplement: S3 Fig — Inhibition of SCD1 in other cell types. A dose response curve of SCD1 inhibition of DENV2 replication in C6/36 cells (A) and A549 (B). Cells were infected with DENV2 (MOI = 0.5) and treated with the indicated concentrations of the SCD1 inhibitor. Virus supernatant was collected at 24hr post infection and quantified by plaque assay. Cytotoxicity was measured by the fluorescence of the reduction of resazurin to resorufin. (TIF) [file ppat.1007261.s005.tif]

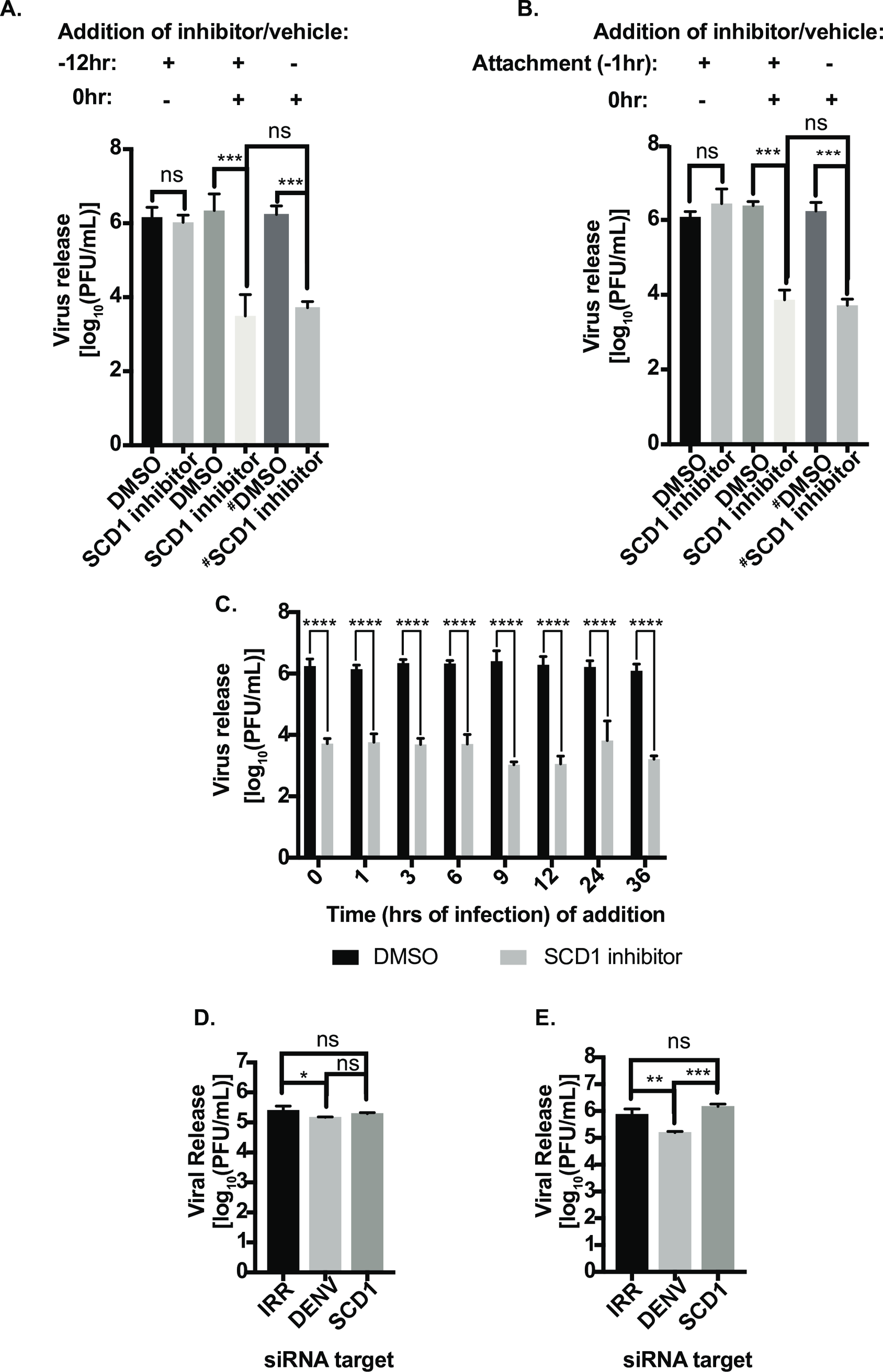

Supplement: S4 Fig — Time of addition of SCD1 inhibitor and siRNA. (A-C) Huh7 cells were infected with DENV2 (MOI = 0.5), overlaid with DMEM, the inhibitor was added at the indicated time points, and virus supernatant was collected at 48hr. (A) The inhibitor was added to cells at 12hr prior to infection and then either removed or kept on for 48hr, or the inhibitor was added after adsorption of the virus (time = 0). (B) The inhibitor was added during the attachment stage and then either removed or retained for 48hr, or the inhibitor was added after adsorption of the virus (time = 0). (C) The inhibitor was added at the indicated timepoints and virus supernatants were collected at 48hr. (D-E) Huh7 cells were infected with DENV2 (MOI = 0.1) and incubated for 24hr. Then the indicated siRNAs were added to the cells. Supernatant was collected and titrated at (D) 48 and (E) 72hr post infection. (ns = not significant, * = p<0.05, ** = p<0.005, *** = p<0.0005, **** = p<0.0001 compared to control, #These virus samples are the same and is shown twice for comparison to the other data). (TIF) [file ppat.1007261.s006.tif]

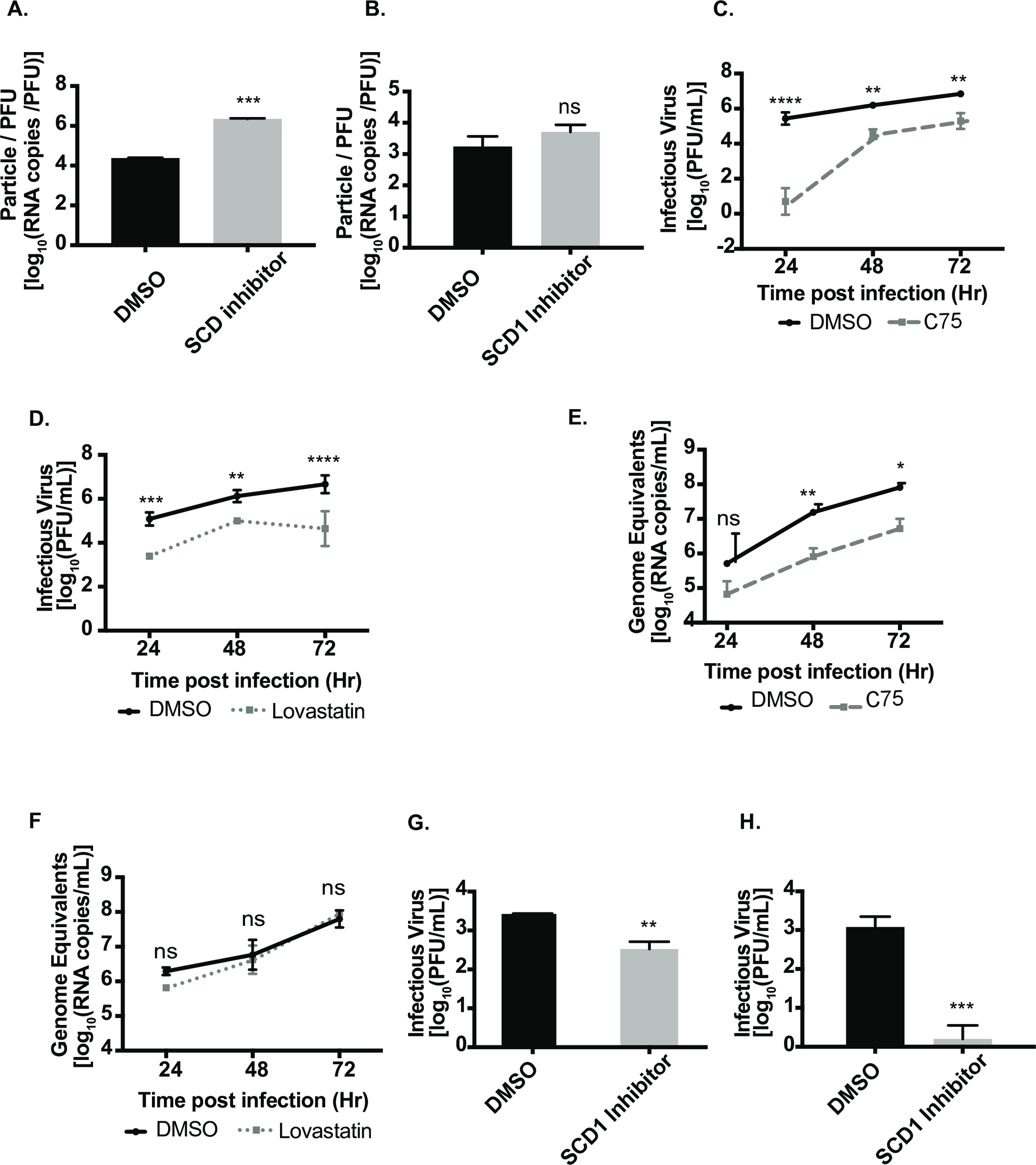

Supplement: S5 Fig — DENV2 treated with SCD1 inhibitor has a defect in infectivity in human cells but not mosquito cells. (A) Huh7 cells were infected with ZIKV and treated with the SCD1 inhibitor for 24hr. Supernatant was collected and quantified by plaque assay. RNA was extracted and genome copies measured by qRT-PCR. (B) C636 cells were infected with DENV2 and treated with the SCD1 inhibitor for 24hr. Supernatant was collected and quantified by plaque assay. RNA was extracted and genome copies measured by qRT-PCR. (C-F) Huh7 cells were infected with DENV2 (MOI = 0.5) and treated with C75 or DMSO (C, E) or Lovastatin or DMSO (D, F). Supernatants were collected at the indicated time points and viral titer determined by plaque assay (C, D) or RNA was extracted and genome equivalents measured by qRT-PCR (E, F). (G-H) Cells were infected with DENV2 (MOI = 3) and treated with 10μM SCD1 inhibitor. This virus was collected at 24hr and subsequently used to re-infect new cells at MOI = 0.1 in the absence of inhibitor. Supernatant was collected at 24hr and viral titer determined by plaque assay. (G). Experiments in Huh7 cells. (H). Experiments in A549 cells. (ns = not significant, * = p<0.05, ** = p<0.005, *** = p<0.0005, **** = p<0.0001 compared to control) (TIF) [file ppat.1007261.s007.tif]

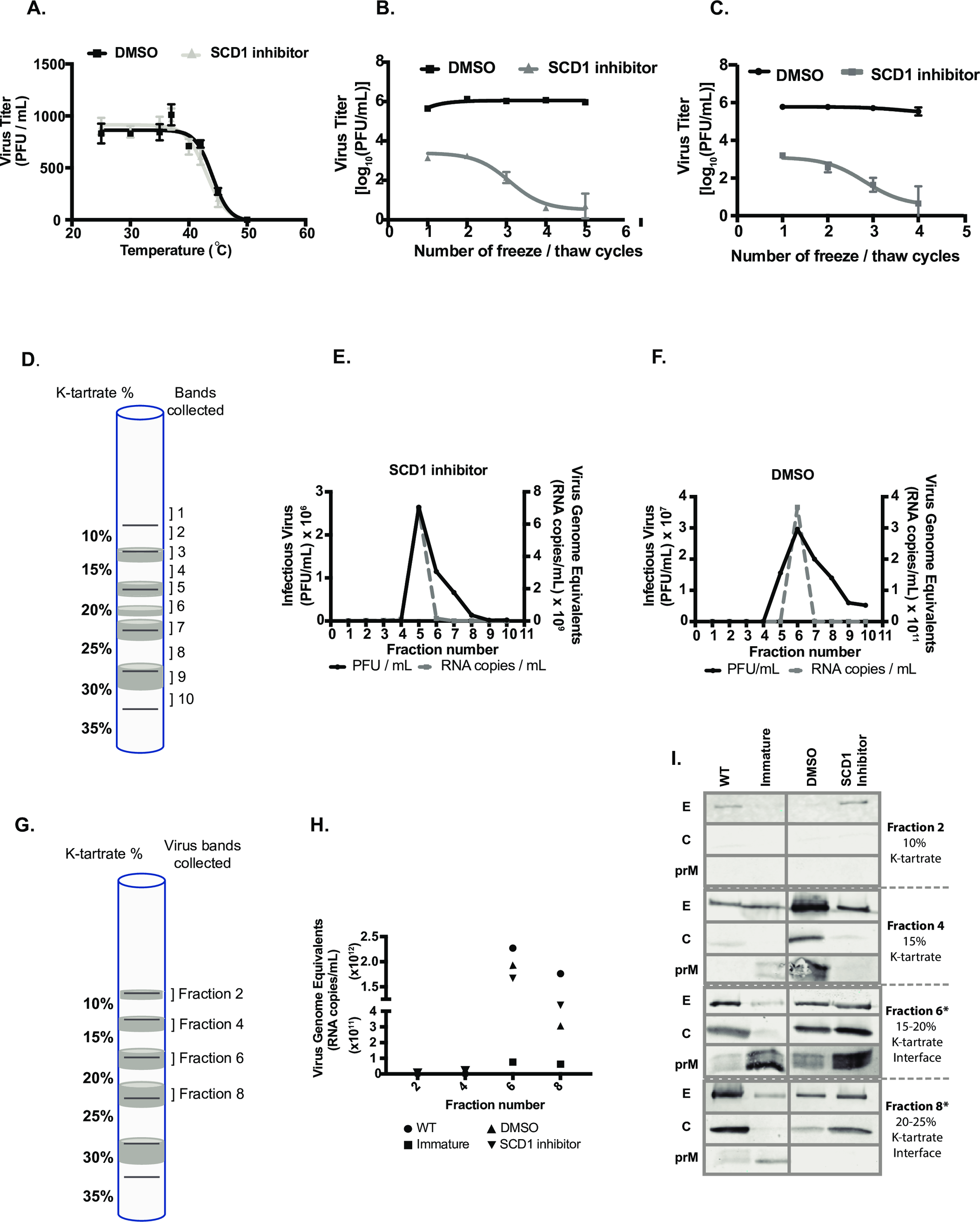

Supplement: S6 Fig — Characterization of virus grown in the presence of SCD1 inhibitor. Huh7 cells were infected with DENV2 and treated with the SCD1 inhibitor or vehicle. Supernatant was collected at 24hr and the viral titer quantified by plaque assay. (A). The virus was then diluted to 1000 pfu/ml and subjected to the indicated temperature for 15 minutes. The virus was allowed to recover at room temperature and then used to infect BHK cells. Plaques were counted and the PFU/mL was calculated. (B-C). The indicated virus samples were subjected to freeze/thaw cycles. Virus was thawed, titrated on BHK cells and returned to -80°C until frozen. (B) DENV2 was cycled through this process 5 times. A linear regression was performed and the control samples yielded a slope of 0.06 that was not significantly different from zero, while the SCD1 inhibitor samples yielded a slop of -0.75 that significantly deviated from zero with p = 0.02. (C) ZIKV was cycled through this process 4 times. (D-I) Huh7 cells were infected with DENV2 and left untreated (WT), treated with 20mM NH4Cl (immature), 10mM SCD1 inhibitor or vehicle (DMSO). (D-F) Virus grown with the SCD1 inhibitor or DMSO was collected at 24hr, concentrated through a sucrose cushion and run on a K-tartrate gradient. (D) Ten fractions were collected (labeled 1–10). Distinct bands observed are shown in grey. The virus in each fraction from DMSO (E) or SCD1 inhibitor treated samples (F) was titrated (black) and RNA from each fraction was extracted to measure genome equivalents (grey). (G-I) Virus supernatant was also collected at 72hr post-infection, PEG precipitated, concentrated through a sucrose cushion, and purified on a K-tartrate gradient. (G) Distinct bands (fractions 2, 4, 6 and 8) where virus was observed (grey) were concentrated, and buffer exchanged. (H) RNA was extracted from these bands to measure genome equivalents and (I) Western blots performed to probe for envelope, capsid and prM viral proteins. *These data (for fractions 6 and 8) [file ppat.1007261.s008.tif]
